# Brain Plasticity and Cell Competition: Immediate Early Genes Are the Focus

**DOI:** 10.3390/cells14020143

**Published:** 2025-01-19

**Authors:** Pavel P. Tregub, Yulia K. Komleva, Maria V. Kukla, Anton S. Averchuk, Anna S. Vetchinova, Natalia A. Rozanova, Sergey N. Illarioshkin, Alla B. Salmina

**Affiliations:** 1 Research Center of Neurology, 125367 Moscow, Russia; 2 I.M. Sechenov First Moscow State Medical University, 119991 Moscow, Russia

**Keywords:** neuroplasticity, cell competition, immediate early genes, Arg3.1/Arc, c-Myc, c-Fos, neurodegeneration, brain aging

## Abstract

Brain plasticity is at the basis of many cognitive functions, including learning and memory. It includes several mechanisms of synaptic and extrasynaptic changes, neurogenesis, and the formation and elimination of synapses. The plasticity of synaptic transmission involves the expression of immediate early genes (IEGs) that regulate neuronal activity, thereby supporting learning and memory. In addition, IEGs are involved in the regulation of brain cells’ metabolism, proliferation, and survival, in the establishment of multicellular ensembles, and, presumably, in cell competition in the tissue. In this review, we analyze the current understanding of the role of IEGs (c-Fos, c-Myc, Arg3.1/Arc) in controlling brain plasticity in physiological and pathological conditions, including brain aging and neurodegeneration. This work might inspire new gene therapy strategies targeting IEGs to regulate synaptic plasticity, and potentially prevent or mitigate neurodegenerative diseases.

## 1. Introduction

One of the attributes of the functional activity of the central nervous system (CNS) is neuroplasticity. This quality determines the ability of the nervous system to adapt to changes in the environment. Thus, in response to stimuli of different origin, numerous changes in the structural and functional parameters of the brain are initiated, including the establishment and elimination of synapses, the remodeling of brain neuronal circuits, the promotion of neurogenesis, the angiogenesis and programmed cell death, the activation of glial cells, changes in dendritic or axonal architecture, the myelination of axons, and the adjustment of local microcirculation to the current needs of active brain regions [1,2].

**Figure 1:**
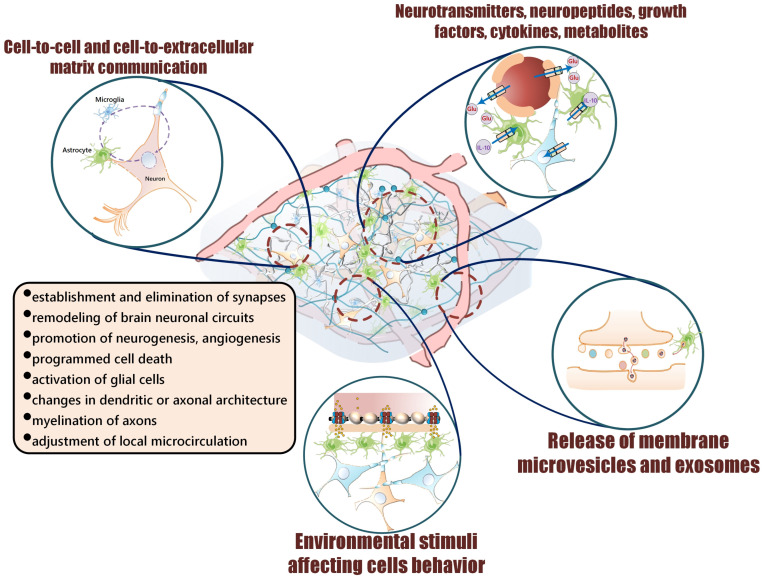
Changes in the structural and functional parameters of the brain occurring through the mechanisms of brain plasticity.

All these events are under the control of (i) neurotransmitters, neuropeptides, gliotransmitters, hormones, growth factors, cytokines, alarmins, metabolites (glucose, lactate, ketone bodies, fatty acids, etc.) released from activated cells or transported from the blood; (ii) cell-to-cell communication and cell-to-extracellular matrix communication; (iii) the release of membrane microvesicles and exosomes from activated or damaged cells; (iv) environmental stimuli affecting cell behavior (Figure 1) [3,4]. As a result, the sensing and processing of any short-term stimulus causes long-term changes in the integral activity of the brain, being responsible for long-term effects in cognition, emotions, social behaviors, and the brain-mediated regulation of the functional activity of peripheral tissues and organs.

It is known that an important functional component of nervous system cells is the expression of immediate early genes (IEGs) that induce plastic changes in the neuronal synapses [5,6]. To date, a large number of IEGs have been identified, and their cellular functions vary considerably [7]. Thus, the activation of these genes is thought to be one of the major evolutionarily conserved components of the neuronal response to injury [8]. IEGs also control the synthesis of regulatory proteins involved in the mechanism of memory consolidation [9]. They participate in many cellular functions, including cell proliferation and differentiation processes [10]. In addition, the overexpression of some IEGs (e.g., c-Fos and zif/268) is involved in the development of neuronal cell apoptosis in neurodegenerative diseases [11].

Brain plasticity is always associated with significant changes in the number and activity of synapses. A prolonged boost of synaptic activity is known as long-term potentiation (LTP), as opposed to long-term depression (LTD), which is a decrease in synaptic transmission strength after a period of neuronal activity. It is generally accepted that LTP and LTD play an important role in memory encoding and learning. There are two stages of LTP: (1) early LTP lasts from 1 to 2 h and is characterized by the expression of IEGs and post-translational modifications of already existing synaptic proteins; (2) late LTP lasts for several hours or even days and is characterized by significant changes in the postsynaptic density (PSD), dendritic spine enlargement, and the establishment of new synapses required for memory consolidation. Late LTP requires the expression of new genes and proteins, and the same process occurs in each round of further memory reconsolidation [12]. LTD is caused by decreased transmission efficacy and is characterized by the elimination of weak synapses and downregulation of AMPA glutamate receptors to support the most effective synaptic plasticity state, thereby contributing to the establishment of memory engrams. The restriction of LTP by LTD in dendritic subcompartments drives this mechanism [12]. As was reported in recent studies [13], LTD should not be considered a process that alters memory, since synapse elimination in dendritic spines during LTD is necessary to produce a memory that represents all details correctly and does not overlap with information noise.

All mechanisms of synaptic plasticity are coupled with short-term and long-term changes in the protein complexes located in pre- and postsynaptic membranes. The establishment and activation of multiprotein platforms provides a substrate for maintaining the activity of an information-perceiving neuron. One of these complexes is the AMPAR glutamate receptor, which is a hetero-tetramer forming a ligand-dependent channel. It allows for Na^+^ to enter the postsynaptic neuron, resulting in depolarization [14]. NMDAR glutamate receptors are both chemo- and voltage-sensitive and have a slow activation profile and long-lasting activity. These receptors allow for Ca^2+^ to enter the cell and are involved in LTP development [15,16]. Metabotropic glutamate receptors, mGluRs, also take part in LTP induction, most likely through an alternative, NMDAR-independent pathway which requires the expression of one IEG—Arc/Arg3.1 [17].

The expression of different glutamate receptor subtypes largely determines the type of response to the action of an excitatory stimuli. In neurons with high NMDAR and low AMPAR expression, the activation of “silent” synapses requires AMPAR externalization through the SNARE (soluble NSF attachment receptor)-controlled exocytosis of vesicles supported by the activity of synaptotagmin 1/7 [18] or complexin [19]. Clathrin-dependent AMPARs endocytosis mediated by Arc/Arg3.1 or PICK1 (protein interacting with C-kinase 1) leads to LTD induction and LTP suppression [20]. It should be noted that the activity of complexin, which binds to a complex of SNARE proteins, is only relevant to exocytosis during the induction of LTP but is not involved in the baseline synaptic activity [19]. The knockout of the Arc/Arg3.1 gene disrupts mGluR-mediated LTD but not NMDA-mediated LTD [17]. It is important to note that mutations in genes encoding SNARE proteins are often associated with a variety of neurological disorders (“SNAREopathies”), including epilepsy, intellectual disability, and neurodevelopmental conditions such as autism spectrum disorders [21,22].

The coordinated and synchronous activation of pre- and postsynaptic neurons is crucial for proper synaptic transmission. For LTP, it is obligatory that both presynaptic and postsynaptic neurons are simultaneously active. It is then that the glutamate-dependent activation of postsynaptic NMDARs becomes possible, thereby resulting in significant cytosolic Ca^2+^ increases and the induction of LTP. In contrast, the repeated activation of presynaptic neurons deprived of the concomitant activity of postsynaptic neurons or lacking a significant increase in cytosolic Ca^2+^ levels results in LTD [20].

Effective synaptic transmission requires the establishment of a multiprotein platform in the receiving neuron, known as a postsynaptic density (PSD). This is a complex multi-layered compartment where critical protein–protein interactions modulate synaptic transmission. Proteins are organized there to ensure the efficient downstream transmission of a signal. The PSD structure differs in its excitatory and inhibitory synapses. The surface layer contains transmembrane proteins such as NMDARs and AMPARs or GABA and glycine receptors. The cytoplasmic layer contains a variety of proteins that interact with the cytoskeleton and produce dynamic changes in the dendritic spine profile. Scaffold proteins are abundantly present within the entire PSD. They regulate signaling pathways and recruit other proteins (e.g., NMDARs and AMPARs) into the PSD complex [23]. Scaffold proteins like PSD95 or filamin A are located at different distances from the postsynaptic membrane, and their distribution, post-translational modifications, and protein–protein interactions are sensitive to the actual synaptic activity. PSD structural integrity and remodeling is controlled by the actin cytoskeleton: dendritic spines are affected by Ca^2+^- and NMDA-dependent dynamic changes in the actin microarchitecture [24,25].

As we have mentioned above, the development of LTP and LTD is coupled with the expression of various genes in neurons, and some of them belong to the group of IEGs [26]. Among the IEGs, c-Fos, Arc/Arg3.1, NPAS4, Egr1 and Nr4a1, c-Myc, etc., are induced as early as possible in LTP and their expression occurs independently of de novo protein synthesis. Another subgroup includes the “second wave” genes (BDNF, Homer1, Nrn1, Rgs2, etc.), which have a slower expression profile [26,27]. The expression of c-Fos, Homer1a, and Arg3.1/Arc genes is triggered by either LTP and LTD [28,29]. Currently, IEGs are considered “signatures” of memory engrams: for instance, hippocampus-mediated learning results in the fast induction of IEGs expression in cortical and hippocampal neurons. Protein products of IEGs expression trigger plasticity-associated changes in neurons. Thus, IEGs often serve as neuronal activity markers, allowing for the detection of engram cells that are believed to hold the memory in the brain [17]. It was experimentally confirmed that the targeted activation of c-Fos-expressing cells (e.g., with opto- or chemogenetic approaches) makes it possible to modify mechanisms involved in memory encoding, storage, and recall [13,30], whereas the inhibition of c-Fos expression in neurons results in LTP and LTD suppression [31].

The activity of other IEGs of the “first wave”, such as Zif268/Egr1 and NPAS4, also determines the strength of newly encoded memories [31], the activation and deactivation of hippocampal neuronal subpopulations [32], the development of altered synaptic plasticity in stressful conditions [33], the efficacy of activity-dependent DNA repair in neurons [34], the establishment of excitation-to-inhibition balance in activated neuronal circuits [35], the regulation of neuronal proteasomal machinery [36], and the induction of experience-triggered neurogenesis [37]. IEGs of the “second wave” contribute to specific changes in activated neuronal cells, including the modulation of G-protein signaling by RGS2 [38], support of PSD establishment, remodeling and relocation by Homer1 and BDNF [39,40], the facilitation of LTD by BDNF [41], and the internalization of AMPA receptors by Homer1 [42]. It should be taken into consideration that the activation of different IEGs may be achieved by various stimuli (membrane depolarization or the action of growth factors), with various dynamics and functional outcomes [42]. However, among all the IEGs, some are particularly interesting because of the relatively high stimulus threshold for the induction of their expression (c-Fos) [43], their ability to be secreted and transferred between activated neurons (Arg3.1/Arc) [44], or their potent regulatory activity in developing neuronal cells (c-Myc) [45].

Progress in studying the activity-dependent transcriptional changes in neuronal cells has led to the development of new approaches to deciphering the molecular mechanisms of learning, memory [46], and the functional mapping of brain cells [47].

The evidence suggests that, in various normal and transformed cells, the proteins encoded by IEGs may have a huge number of other biological effects [48,49]. For example, the expression of IEGs affects cells growth and proliferation, cell-to-cell signaling, the transport of metabolites, energy production, DNA repair, and cell cycle progression [50]. In the brain, synaptic plasticity may trigger some long-lasting events, affecting the fate of cells involved in synaptic plasticity. Indeed, an increase in the expression of IEGs (c-Fos, Arg3,1/Arc) in stimulated hippocampal neurons was found in the cells with most prominent changes induced by learning; this is critical for memory consolidation [51]. Such changes may include the appearance of new dendritic spines [52], the dynamic remodeling of PSD associated with an altered pattern of protein phosphorylation [53], the suppression of apoptosis and increased neuronal survival [54], and extensive synaptic pruning by apoptosis-like mechanisms in neurons [55]. Thus, it is tempting to speculate that such plastic changes in the brain, induced by learning or memory encoding, could be coupled with the induction of mechanisms resembling the phenomenon of cell competition, where suboptimal cells are removed in favor of cells with higher “fitness” [56].

However, the logics of engram establishment and maintenance—in which neuronal fate is adjusted to increase the efficiency of neural circuits [56]—suggest that neuronal cell competition might be predominantly linked to synaptic competition, or competition between proliferating neuronal progenitors whose development in neurogenic niches is driven by learning [57]. Recent experimental findings, obtained in vivo with c-Fos as a marker of recently activated neurons, support this idea: activity-dependent synaptic plasticity results in the selection of neurons for memory encoding, and the selection process is competitive rather than autonomous of cells [58].

The main goal of this review is to summarize the data on the possible role of some IEGs—c-Fos and Arg3.1/Arc, which are differently induced in active postsynaptic neurons, and c-Myc, whose expression mainly drives the long-lasting mechanisms of brain plasticity—in the regulation of intercellular communication and cell competition in (patho)physiological conditions, including brain aging and neurodegeneration.

## 2. c-Fos, Arg3.1/Arc, and c-Myc as Potent Regulators of Brain Plasticity

### 2.1. c-Fos Gene

The c-Fos gene was initially discovered as a cellular homolog of the oncogene v-Fos and was later found to affect stimulus-transcription coupling in neurons [59]. The Fos gene family consists of four members: FOS, FOSB, FOSL1, and FOSL2 [60]. These genes encode leucine zipper proteins that can dimerize with JUN family proteins to form the AP-1 transcription factor complex. Thus, FOS proteins have been linked to regulators of cell proliferation, differentiation, and transformation. In some cases, FOS gene expression is also associated with apoptotic cell death. FOS is expressed in a Ca^2+^- and CREB-dependent manner in recently activated neurons that also serve as engram cells to encode long-term memories [61,62]. In dissociated mouse dorsal root ganglion neurons, differences in c-Fos activation did not correlate with a peak in intracellular Ca^2+^, but in the case of effective stimulation, a higher increase in intracellular Ca^2+^ concentrations was required for the stimulus-dependent expression of the IEGs [63].

c-Fos expression rapidly and transiently increases during the depolarization of neurons; then, the c-Fos protein heterodimerizes with members of the JUN family of proteins (c-Jun, JunB, and JunD). As a result, the transcription factor activator protein 1 (AP-1) is formed to transform short-term stimuli into a long-term neuronal response [64]. c-Fos forms a tight but non-covalently bound complex with the transcription factor JUN/AP-1 [65]. In the heterodimer, the major sites of FOS and JUN/AP-1 appear to interact with symmetrical DNA half-sites. Upon TGF-beta activation, a multimeric SMAD3/SMAD4/JUN/FOS complex forms at the AP1/SMAD binding site to regulate TGF-beta-mediated signaling [66]. This serves a critical function in the regulation of cell development for skeletal formation and maintenance. Later, these protein complexes are degraded via the activity of the ubiquitin–proteasome system [67].

Transcription factor CREB controls the transcription of genes with a CRE site (including c-Fos). Long-term synaptic plasticity is associated with neurons that activate the CREB/c-Fos system via an NMDAR-dependent mechanism [68]. The rapid dynamics of c-Fos transcription patterns were demonstrated after various kinds of brain stimulation [69,70]. For instance, in the hippocampus, c-Fos expression was elevated 10-fold within 20 min after stimulation (in a similar manner as Arg3.1/Arc expression) [49]. In the visual cortex, the expression of c-Fos peaked 24 min after stimulation and disappeared after 240 min [71]. The optogenetic stimulation of glutamatergic neurons in the prefrontal cortex resulted in elevated c-Fos expression not only in this brain region but also in the functionally connected ones, including the hippocampus and perirhinal cortex, 30 min after light delivery in vivo [72].

c-Fos expression is differentially regulated in active neurons. For instance, after inhibitory avoidance training, c-Fos in the hippocampus was evident within 1 hr and the longer-term post-training period (24 h), maintaining traces of the memory [73]. The functional pERK-dependent coupling and cycling of c-Fos and CREB expression are characteristic of engram neurons that store the information via olfactory stimuli [61,74].

The ∆FosB transcription factor, another member of Fos family that dimerizes with JUN to form the AP-1 complex and may further accumulate due to its high stability, is therefore responsible for the development of sensitization to chronic drug exposure, affecting numerous intracellular mechanisms controlled by cyclin-dependent kinases and NF-kB [75]. Since FosB and its splice variant, ΔfosB, have delayed activation and persist longer than c-Fos, they might be considered markers of chronic neuronal activation [75,76].

Recent data confirm that c-Fos expression in the postnatal developing mouse brain serves as a marker of maturity in the neuronal circuitry in the hippocampus and some other brain regions during wakefulness. There is a basal level of c-Fos expression in the absence of sensory stimulation or the application of specific behavioral tasks [77].

c-Fos expression is not exclusive to neurons: actively proliferating brain cells (microglia, astrocytes, and oligodendrocytes) utilize it for activation in proinflammatory conditions [78].

Thus, c-Fos is considered to be an important factor of brain homeostasis [79,80] and is a marker of the stimulus-dependent transcriptional activation of neurons, as was excellently reviewed in [42,46,81,82]. The altered expression of c-Fos is linked to the aberrant excitability of neurons. When c-Fos expression was suppressed in the hippocampus of knockout mice, more severe seizures caused by kainic acid, an increase in the excitability of neurons, and the death of neural cells were observed [80]. Mice lacking c-Fos expression in the brain show normal behavior and impairments in the hippocampus-dependent spatial and associative learning tasks, as well as reduced LTP in hippocampal CA3-CA1 synapses [83].

### 2.2. Arg3.1/Arc Gene

The Arg3.1/Arc gene (Activity-Regulated Cytoskeleton-associated protein, Arg3.1 and KIAA0278) was discovered while searching for IEGs that responded to neuronal activity in cortical and hippocampal glutamatergic neurons [84]. The gene product, the Arg3.1/Arc protein (45 kDa) [85], is considered to be an obligatory participant in the molecular machinery necessary for learning and memory: the suppression of Arg3.1/Arc expression leads to impairments in long-term, but not short-term, memory [86]. The Arg3.1/Arc protein is highly conserved in mammals, birds, reptiles, and amphibians, but is absent in fish [87,88]. Arg3.1/Arc encodes proteins which allow for the functional “tuning” of the activated neuron through some of the following mechanisms [86,87]: the regulation of actin dynamics, which is important for the consolidation of long-term potentiation; the endocytosis of AMPA receptors, which promotes the conversion of early long-term potentiation to late potentiation; chromatin remodeling, which is important for epigenetic regulation; the stimulation of the proteolytic degradation of APPs (amyloid precursor protein) and Notch1 proteins, which modulates the expression genes due to the appearance of intracellular fragments of these proteins in cells; interactions with the postsynaptic density protein PSD95. In neuronal cells, the activation of NMDARs, mGluRs, TrkB, and mAChRs triggers the activity of numerous protein kinases, including ERK, PKA, and PKC, which, in turn, activate the transcription of the Arg3.1/Arc gene [89]. The phosphorylation of TCF, which binds to several regions of the SRE sequence in the Arg3.1/Arc promoter, significantly increases the transcription of Arg3.1/Arc gene [4]. In general, neuronal stimuli cause the rapid (within 5 min) transcription of the Arg3.1/Arc gene and translocation (within 30 min) of its mRNA from the nucleus to the cytoplasm [90]. Then, Arg3.1/Arc mRNA is translated in the soma and activated dendrites, and its translation is downregulated via the FMRP protein. In addition, mGluRs stimulation triggers the rapid translation of pre-existing Arg3.1/Arc mRNAs in dendrites [91,92]. The polysomal translation of Arg3.1/Arc mRNA generates a burst of protein synthesis that accompanies an increase in the AMPARs endocytosis. Then, the newly synthesized protein is rapidly localized close to the active synapses and promotes mGluR-mediated LTD [93].

Arc/Agr3.1 expression in neurons is induced within a few minutes and continues through the first 2-4 h of LTP, along with changes in AMPARs expression and actin cytoskeleton remodeling [94]. Upon LTP induction, a fivefold increase in AMPARs exocytosis occurs in dendritic spines [95] whose structural plasticity is under the control of Arc/Arg3.1-mediated mechanisms [96,97]. It is generally accepted that Arg3.1/Arc translation coordinates mGluR-driven LTD induction, at least in experience-driven brain plasticity [98]. The activation of mGluR-expressing neurons in the CA1 region of mouse hippocampus in vivo leads to LTD induction coupled with an increase in Arg3.1/Arc expression, its accumulation in dendrites, the downregulation of AMPARs expression, and the weakening of excitatory synapses [98]. Arc-stimulated AMPARs endocytosis is particularly associated with dendritic spine remodeling (an increase in the number of thin, but not mushroom-shaped, spines) that prevents spontaneous aberrant neuronal activity [96]. This process seems to be involved in the so-called “heterosynaptic plasticity” and mutual interference of active and inactive synapses via differential Arg3.1/Arc expression or, more probably, via intercellular Arg3.1/Arc transfer [99,100].

The mechanism of Arc-driven synaptic control is not yet fully understood, although there is strong evidence that it affects LTP through regulating the dynamics of actin cytoskeleton [101] or clathrin-mediated AMPARs endocytosis [102,103]. However, elevated levels of Arg3.1/Arc proteins are needed for both LTP and LTD and the reason for this might be the regulation of PSD phase separation [104]. The rapid Arg3.1/Arc expression observed after LTP and LTD induction interrupts the interaction with PSD95 and promotes PSD remodeling, while Arg3.1/Arc degradation is required to establish a new stable state in the PSD platform [104].

However, the abnormal persistence of the Arg3.1/Arc protein due to the inhibition of its proteasomal degradation in neurons alters their Arc-driven signaling pathways and enhances the mGluR-induced LTD associated with learning impairments [105]. Using Arg3.1/Arc knockout mice, Kyrke-Smith, M. et al. showed that Arg3.1/Arc is not required to maintain hippocampal LTP and may instead act in heterosynaptic plasticity and epigenetic processes [106]. However, recent studies have shown that persistent Arg3.1/Arc expression regulates long-term potentiation magnitude and metaplasticity in the CA1 of the hippocampus in ArcKR mice [107]. The latter is possible since interactions between Arg3.1/Arc and Tip60 histone acetyltransferase lead to increased H4K12 acetylation and promote the expression of a number of genes [108]. It is noteworthy that the reduced activity of Tip60 leads to alterations in the expression of genes encoding for proteins active in inflammation, cell cycle control, and learning [109]. In the nucleus, the Arg3.1/Arc protein interacts with the βSpIVΣ5 (beta-spectrin IV) protein. The association between Arg3.1/Arc and βSpIVΣ5 leads to the appearance of nuclear bodies (PML-NB) in the hippocampal neurons in vitro. Since PML-NB are macromolecular domains that are capable of regulating gene transcription [110], Arg3.1/Arc may serve as a transcriptional regulator [111].

**Figure 2:**
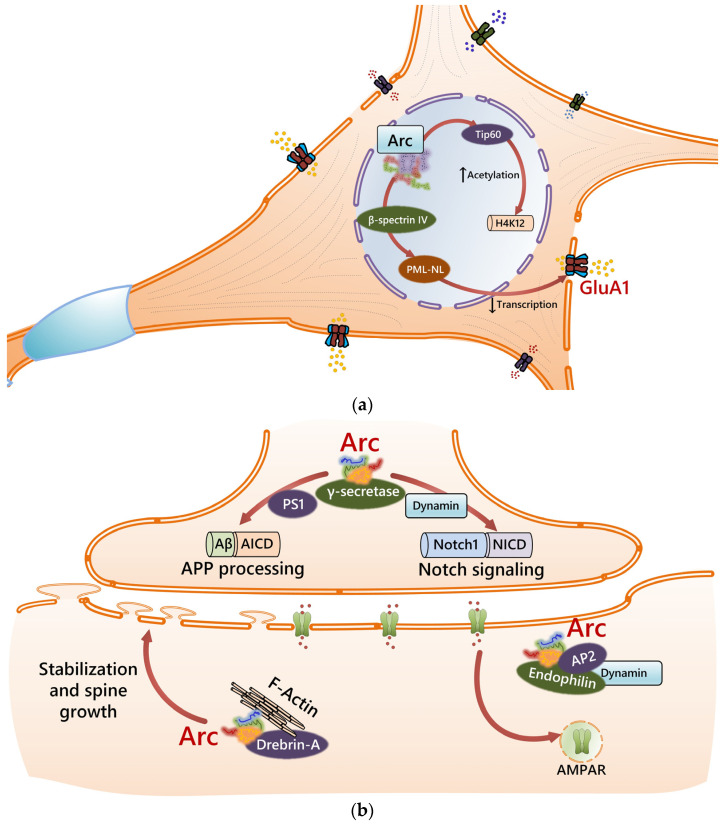
Interactions with various cellular proteins mediate Arg3.1/Arc functions in the nucleus (**top**) and synapses (**down**) associated with LTP and LTD. Note: (**a**) The interaction between Arc and histone acetyltransferase Tip60 leads to an increase in the acetylation of histone H4K12 and promotes the expression of a number of genes. In addition, in the nucleus, the Arc protein interacts with the beta-spectrin IV isoform, which leads to an increase in nuclear cells characteristic of promyelocytic leukemia (PML-NB), which are capable of regulating the transcription of GluA1 genes. (**b**) Arc regulates synaptic activity through enhancing clathrin-mediated endocytosis AMPAR. The Arc complex with dynamin positively regulates the gamma–secretase-mediated cleavage of Notch1 in neurons and leads to an increase in the production of intracellular fragments—NICD. The activity-dependent generation of β-amyloid and AICD requires the expression of Arc, which, together with the presenilin protein (PS1), allows for the localization of γ-secretase in dendritic endosomes for the proteolysis of ARP.

In sum, the key characteristics of Arg3.1/Arc’s effects in activated neurons are shown in Figure 2.

### 2.3. c-Myc Gene

The c-Myc gene, like c-Fos, was initially described as a protooncogene [112]. Its increased expression in various cells is combined with a lack of response to physiological signals, leading to cell transformation and tumor development [113]. Therefore, most of the data on c-Myc are linked to its role in the development of tumors [114,115], but there is accumulating evidence of its role in normal cell growth and proliferation, survival, and metabolism, as well as in vascular permeability control and vessel remodeling [116,117]. According to recent data, c-Myc is a regulator of immunological memory and immune tolerance [118].

Three exons of the c-Myc oncogene encode a protein sequence of 439 amino acids with several conserved sites that are similar in all members of the family (c-Myc, l-Myc, and n-Myc) [119], which act as transcription factors [120]. The Max protein (Myc-associated factor X) accompanies Myc during dimerization, and they bind to DNA with non-absolute specificity [121]. Myc-Max dimers form the basis for the binding of other transcription factors, such as Miz–1 (Msx-interacting zinc enzyme) and Skp2 (protein 2 associated with S-phase kinase) [122]. Together with related factors, Myc attracts other transcription regulators, creating a transcription platform with great potential for control [123].

The Myc protein belongs to the category of enhancers, which have many regulatory and amplification factors and control gene transcription through three-dimensional physical interactions with gene promoters, regardless of their position and orientation relative to their site at the beginning of transcription [115,124].

Extracellular signal-regulated kinase (ERK)-mediated phosphorylation increases the stability of c-Myc in a similar manner to c-Fos or Egr1 phosphorylation mediated by ERK; therefore, it was suggested that c-Fos, Egr1, and c-Myc may serve as ERK sensors in activated cells [79,125]. It should be noted that the ERK signaling cascade is involved in the regulation of PSD multiprotein machinery in postsynaptic neurons [126],

In contrast to c-Fos or Arg3.1/Arc, c-Myc expression is not directly linked to synaptic activity but might be important for long-lasting changes in the metabolism and viability of cells. In the developing and adult brain, the expression of c-Myc controls events that are more related to cell proliferation (e.g., in neurogenesis) and metabolism control [127,128], whereas the altered expression of this gene can provoke the mechanisms of brain aging [129,130]. Thus, unlike actively proliferating cells of the body, the overexpression of c-Myc in neurons leads to the death of neuronal cells and the subsequent development of a neurodegenerative phenotype [129,131]. At the same time, the expression of c-Myc has a positive effect following stroke and traumatic damage [132,133].

Thus, c-Myc expression has a multidirectional effect in neuronal cells at different stages of differentiation and in different phases of the cell cycle, which is especially important for neurogenesis in the embryonic and postnatal periods, as well as for the proliferation and activation of glial cells. The overexpression of c-Myc induces mitochondrial remodeling, increases proliferation, and reduces the number of neural stem cells in the G0 phase, whereas the knockdown of c-Myc causes cell cycle arrest and increases the number of cells in the G0 phase of the cell cycle [134,135]. A significant effect of c-Myc in neural stem cells is the coordination of metabolism (due to changes in mitochondrial dynamics) and the cell cycle [136].

Since stressful factors, e.g., beta-amyloid oligomers, also induce cell cycle re-entry [137], and the early induction of c-Myc in amyloid-affected cells results in their death, c-Myc, as one of the IEGs, might not directly contribute to the activation of neurons in physiological conditions, but could regulate the elimination of damaged postmitotic neurons. c-Myc-immunopositive neurons and hyperphosphorylated c-Myc in neurons and glial cells have been detected in the loci of brain ischemia and neuroinflammation [131,138]. The elevated expression of c-Myc in dorsal root ganglion neurons prevents axonal degeneration in models of spinal cord injury, presumably due to the c-Myc-driven transcription of numerous regeneration-associated genes [139]. Moreover, since the expression of c-Myc in mammalian cells is regulated by external stimuli, e.g., growth factors or cell cycle transitions, in activated cells, the elevated expression of c-Myc might be considered a sign of IEG activation [136].

## 3. IEGs and Establishment of Multicellular Ensembles in the Brain

### 3.1. c-Fos Gene

The transition from a short-term response to long-lasting changes in the brain is the key characteristic of brain plasticity. Therefore, the establishment of well-coordinated multicellular neuronal ensembles might be considered a real goal of the complex events underlying synaptic activity. Recently, it was confirmed that new cells in the recall engram in the hippocampus are not added randomly during maturation but differ according to their connections [140].

Some studies suggest that the high c-Fos expression in stimulated neurons makes them active in a well-coordinated manner, which is important for the stable, long-lasting plasticity in the hippocampus [141]. Neuronal ensembles may simultaneously express various IEGs (c-Fos, Arc, and Nr4a1) while encoding to coordinate the activity of cells that are integrated into the memory trace [71,142]. However, it should be noted that c-Fos expression in the hippocampus under conditions of multi-day training might be rather unstable, and increases in the precision of spatial memory do not produce more stable patterns of expression. The optogenetic inhibition of hippocampal neurons that initially express c-Fos after stimulation can affect later memory performance [143].

The idea that c-Fos is involved in the coordination of multicellular ensembles is evidenced by the fact that data on the expression of this IEG show that it is expressed not only by neurons, but also by glial cells (astrocytes, oligodendrocytes, and microglia) [78]. It has also been reported that learning activates c-Fos in hippocampal astrocytes [144].

In mice performing hippocampus-dependent spatial learning tasks, neurons with high c-Fos expression were found in ensembles of cells with highly correlated activity and better spatial selectivity and stability compared with nearby c-Fos-negative cells [141]. The suppression of c-Fos and Arc3.1/Arc expression in engram cells resulted in a loss of memory retrieval, thereby supporting the idea that the re-expression of IEGs in engram cells is required for the reconsolidation of memory and stability of neuronal ensembles [42].

In addition to increasing the expression of c-Fos by the hippocampal neurons during memory formation, there is evidence of the increased expression of c-Fos in neurons in other areas of the brain when exposed to learning stimuli. Thus, c-Fos expression is increased in activated neurons of the parietal cortex in the process of acquiring and extracting memories associated with fear in mice [145]. At the same time, it was shown that c-Fos expression increases after multimodal stress in neurons of the paraventricular nucleus of the hypothalamus [146]. Also, an immunohistochemical mapping of brain slices of mice exposed to a new and familiar environment revealed a significant increase in neural activity involving c-Fos expression in the dentate gyrus, which led to increased exploratory behavior [147].

Recent studies support the idea that the neurons within an engram may have different levels of IEGs, particularly c-Fos expression: c-Fos+ and c-Fos- neurons may control the hippocampal memory index and spatial components of the memory trace, respectively [148]. Thus, a c-Fos-mediated variable functionality of engram cells in encoding the information has been suggested [148,149]. In mice that completed a spatial navigation task, the higher expression of c-Fos was a feature of neurons with more stable tuning over a period of days than those without c-Fos expression; therefore, c-Fos+ neurons contribute to hippocampus-dependent spatial learning [141,150]. This functional selection might be partially supported by the predominant expression of different IEGs [148]. Indeed, in the mouse dentate gyrus, c-Fos-dependent neuronal ensembles support memory generalization, whereas Npas4-dependent neuronal ensembles promote memory discrimination. Thus, functionally distinct neurons controlled by different IEG expression levels might exist within a memory engram [151].

**Figure 3:**
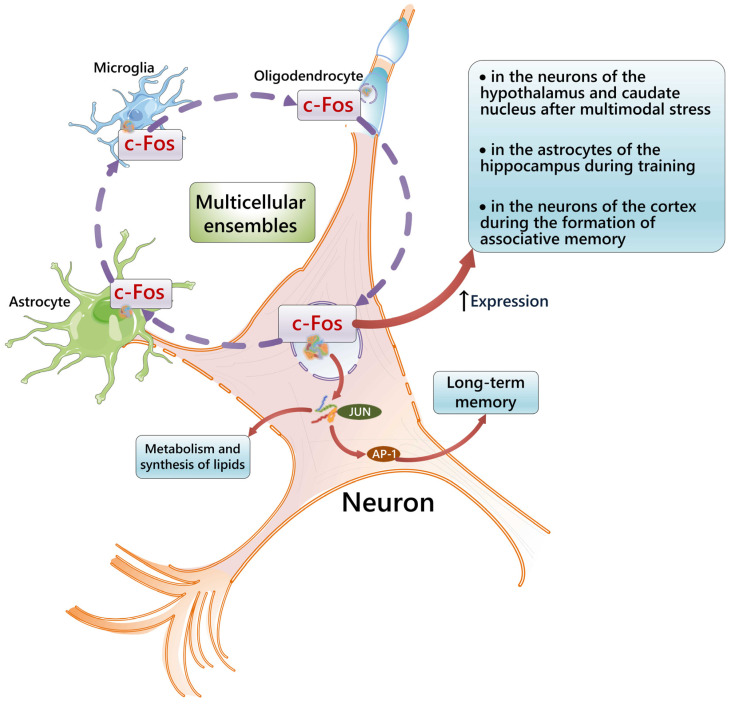
The expression of c-Fos increases with the depolarization of neurons, after which the protein heterodimerizes with proteins of the JUN family and forms a protein activator of transcription factor 1 (AP-1), which converts short-term stimuli into a long-term response. c-Fos also controls the metabolism and synthesis of lipids in connection with EPR. c-Fos expression is found in glial cells (astrocytes, oligodendrocytes, and microglia). Cognitive learning stimuli activate c-Fos expression in hippocampal astrocytes. Activated cortical neurons express c-Fos during the formation of associative memory, and neurons of the hypothalamus and caudate nucleus express c-Fos after exposure to multimodal stress.

The above data show that c-Fos may act as a modulator of the coordinated activity of neurons and accessory cells in learning and memory. Figure 3 shows the possible role of c-Fos in the establishment of multicellular ensembles in brain tissue undergoing plastic changes.

### 3.2. Arg3.1/Arc Gene

The activity of Arg3.1/Arc might be connected with the ability to maintain the stable intercellular communications underlying the establishment of engrams or the integration of newly generated neurons into pre-existing neuronal circuits. What is the possible mechanism of Arg3.1/Arc action within multicellular neuronal ensembles?

Under the conditions of LTP induction in vitro, Arc-positive neurons are likely to be closely located to each other, and Arc expression is high in neurons with strongly modulated correlated activity. Thus, it was suggested that the expression of Arc and c-Fos provides a combinatorial code for distinguishing the subpopulations of neurons that demonstrate different types of activity-dependent plasticity [152].

Phylogenetic sequence analysis reveals that Arg3.1/Arc originates from the Ty3/gypsy family of retrotransposons that are present in the animal, plant, and fungal kingdoms [19,153]. The Arg3.1/Arc gene has structural elements similar to viral Gag (retrotransposon family): its mRNA contains an IRES (internal ribosome entry site) sequence, which ensures cap-independent translation, and the Arg3.1/Arc gene-promoter is adjacent to the SARE sequence (synaptic activity-responsive element) [154,155]. The mechanism of the intercellular transfer of Arg3.1/Arc mRNA within the virus-like protein capsids formed by the Arg3.1/Arc protein itself, discovered several years ago, made it possible, for the first time, to draw an analogy between the mechanisms of cell activation and viral invasion [3,4]. Since it is known that neurons that fire together remain integrated within the neural network (fire together, wire together), the transfer of the mRNA of this gene to other neurons, with subsequent translation into them, may mean that the presence of an IRES in the structure of the mRNA allows for translation even under conditions of cellular stress [156].

Thus, the Arg3.1/Arc protein is able to self-oligomerize and form virus-like capsids that encapsulate RNA, thereby suggesting that Arg3.1/Arc can transmit information across the synapse due to the mechanism of Arg3.1/Arc mRNA translocation between neurons or other cells [153,157]. The drosophila Arc1 protein forms capsid-like structures that bind to a specific retrovirus-like region in the 3′ UTR of its own transcript. This Arc1-Arc1 mRNA complex is loaded into extracellular particles and transferred from neurons to myocytes [157]. Arc capsids contain 5–8 nm spikes protruding from the capsid that are possibly necessary for fusion with the membrane of a recipient cell [158].

Interactions with various proteins and the post-translational modifications of Arg3.1/Arc are highly dynamic and, as discussed above, are controlled by neuronal activity [159,160,161,162]. These events are crucial for the formation of capsids. Interactions with NMDARs initiate the formation of the Arg3.1/Arc capsid by inhibiting protein oligomerization via direct binding to the C-domain [163]. The phosphorylation of Arg3.1/Arc at serine 260 via CaMKIIα regulates its oligomerization into virion-like capsids [93]. Palmitoylation at the N-terminal domain contributes to the fixation of Arg3.1/Arc in the neuronal membrane due to its direct insertion into the hydrophobic core of the lipid bilayer [164]. Arg3.1/Arc does not form a capsid when the purified protein is devoid of nucleic acid, suggesting that the nucleic acid itself is the primer for capsid formation. Specific binding sites in the mRNA, encoded in the 3′ UTR and/or 5′ UTR, allow for more copies of Arg3.1/Arc to be in close contact, thereby causing capsid formation [157]. It might be hypothesized that the virus-like properties of Arg3.1/Arc compete with its ability to bind to postsynaptic density proteins or to induce the endocytosis of AMPARs.

Purified Arg3.1/Arc capsids are also capable of delivering RNA to neurons: virus-like particles with encapsulated Arg3.1/Arc mRNA are released from neurons, e.g., in the form of extracellular vesicles [165]. Presumably, Arg3.1/Arc promotes both the endocytosis and release of extracellular vesicles due to its ability to bind to phospholipids and induce changes at the membrane–cytoskeleton contact points [165]. It is possible that Arg3.1/Arc requires envelope proteins of human endogenous retroviruses for its attachment and entry into recipient cells [166].

It is interesting that in addition to their own mRNA, Arg3.1/Arc capsids can carry small nucleic acids, less than 300 nucleotides in length, including some tRNAs, rRNAs, and microRNAs. Since neurons express a high number of miRNAs, Arc-mediated transfer between neurons, or neurons and glia, can affect many events in activated brain regions [167]. It was proposed that some partner proteins that regulate the synaptic activity and function of neurons, including PSD95, TARPγ2, and CaMKII, are transferred to neighboring neurons via delivery within the Arg3.1/Arc capsids.

It is possible that TRIM5α (Rhesus protein serving as a restriction factor that affects capsids’ disassembly) and Staufen (mRNA-binding protein required for the synapse-targeted delivery of mRNA in neurons with LTP, as well as a regulator of Gag oligomerization and RNA encapsidation in retrovirus-infected cells) are involved in the control of the Arg3.1/Arc life cycle in recipient cells [4]. The monosomal or polysomal translation of large ribonucleoproteins containing Arg3.1/Arc mRNA has been detected in mGluR-stimulated neurons to generate steady-state or burst levels of Arg3.1/Arc proteins in spines and to decrease synapses’ sensitivity [168].

Since the main delivery cargo is mRNA, it is possible that this is immediately translated in the cytoplasm due to the presence of IRES [169]. The presence of IRES allows for the cap-independent translation of eukaryotic and viral mRNA. IRES are responsible for landing ribosomes on both capped and non-capped transcripts in cases where cap-dependent translation initiation is inhibited (e.g., during stress, at a certain stage of the cell cycle, or during apoptosis), which ensures the continuous synthesis of the necessary proteins. For instance, c-Myc, APAF1, and Bcl-2 mRNAs are expressed at low levels under normal conditions, but in stressful situations, their expression is significantly increased due to IRES-dependent translation [154]. Viral IRESs differ from cellular IRESs in highly ordered secondary or tertiary structures, and IRES-supported translation requires special translation factors known as ITAF (IRES trans-acting factors), which help the RNA adopt the correct conformation, ensuring that it is suitable for binding to the 40S subunit of the ribosome. Due to the lack of assembly of the pre-initiator protein complex and several IRES, viruses are reproduced faster [155]. Presumably, a high rate of translation of viral mRNA and Arg3.1/Arc mRNA in recipient cells is necessary to prevent their interaction with TLR7 and TLR8 proteins on the surface of cell endosomes, which aim to recognize exogenous single-stranded RNAs and to promote immune response [170].

Non-neuronal cells in the brain might also receive Arg3.1/Arc from activated neurons: experiments with shRNA-Arc confirmed that an increase in the level of Arg3.1/Arc in astrocytes is not associated with its endogenous production but occurs due to uptake by glial cells [159]. In mixed culture, at high levels of Arg3.1/Arc expression caused by a combination of pharmacological agents simulating LTP (4 BF cocktail containing a mixture of 4-aminopyridine, bicuculline, and forskolin), the transfer of the Arg3.1/Arc protein from neurons to astrocytes was evident [160,161]. Thus, the cell-to-cell transport of Arg3.1/Arc mRNA might play a role in cell-to-cell communication, but the real contribution of this mechanism to the control of synaptic activity, memory consolidation, experience-driven neurogenesis, and the activation of glial cells remains unclear.

**Figure 4:**
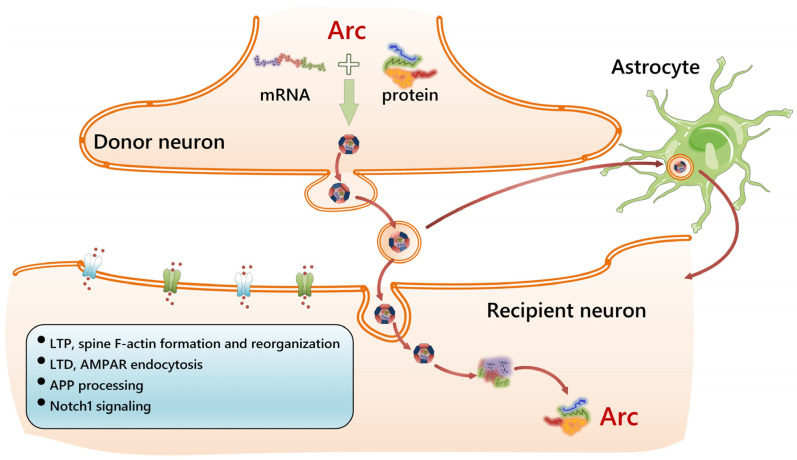
Intercellular Arg3.1/Arc transfer: key targets of action in the recipient cell. Intracellular mRNA transfer leads to its translation at target sites in dendrites, which leads to a high concentration of Arc, supporting capsid assembly at these sites and the encapsulation of Arc mRNA localized in dendrites, which can then be transported as part of the extracellular particles to neighboring neurons, astrocytes, and microglia.

Figure 4 summarizes some data on the intercellular transfer of Arg3.1/Arc in the brain.

## 4. Brain Plasticity as a Phenomenon of Cell Competition: Is There Any Role for IEGs?

Cell competition is a phenomenon that has become a research focus in recent decades. It closely relates to collective cell behavior, representing a mutually coordinated type of intercellular communication. During this process, some cells “recognize” others that are different in their genetic, metabolic, adhesive, or other characteristics, and try to eliminate them in order to maintain a well-balanced population in vivo or in vitro [171]. Levi-Montalcini R. and Hamburger V. were among the first to study cellular competition, showing competition among spinal ganglia neurons for growth factors [172,173]. Cell competition is based on the existence of at least two big subpopulations of cells within the tissue: “winners” (optimal cells with an advantage in their proliferation and development) and “losers” (viable but suboptimal cells). For instance, in *Drosophila melanogaster*, Rp+/+ cells induce caspase-dependent apoptosis in the neighboring Rp+/− cells [174]. c-Myc-overexpressing cells always acquire the “winner” phenotype, and they induce apoptosis or autophagy in the “losers” [174]. Proliferating cells with the “loser” phenotype may also be pushed to terminal differentiation, whereas, for all cell types, forced senescence could be considered another way to be defeated in the cell competition [174]. In many species, the Flower gene is transcribed in a specific isoform and the protein that is most expressed on the cell membrane marks the cell as either a “winner” or a “loser”.

In various organisms and cells, numerous intracellular signaling pathways are modified in cells undergoing cell competition [174]. In general, the “winner” phenotype is associated with the higher expression of c-Myc, JAK-STAT, Wnt, p53, Topo I, BMP, and MAPK. Moreover, c-Myc-overexpressing cells are often considered super-competitors, whereas the “loser’ phenotype is seen in cells with a lower expression of c-Myc, BMP, p53, JAK-STAT, and ERK [171,175,176,177,178]. For instance, the c-Jun N-terminal kinase (JNK) is required to induce apoptosis in loser cells [179]. The protein azot is upregulated in loser cells subjected to elimination due to the presence of c-Myc-overexpressing cells in the tissue [179]. Higher ERK activity prevents cell death due to the development of transient resistance to apoptosis [180]. At the same time, the regulation of cellular competition under the influence of some IEGs can be controlled at the post-translational level: for example, the activity of ERK is determined by its phosphorylation [181].

In the brain, cell competition has specific characteristics:

(i). Due to the high level of brain plasticity, cell competition might have different mechanisms and outcomes in the developing, mature, and aging brain, as well as in the damaged brain and under stimulated/non-stimulated conditions.
(ii). There is a basis for the competitive behavior of synapses that are intrinsically different in their strength and contribution to the development of LTP and LTD, particularly in learning and memory encoding or consolidation. Molecular mechanisms may be involved, which could lead to the selection of subpopulations of cells encoding and storing information [88,182].
(iii). Stem and progenitor cells in neurogenic niches might have higher dependence on the cell competition mechanisms compared to mature neurons.
(iv). In pathological conditions, e.g., neurodegeneration, the removal of mature neurons damaged by the accumulation of aberrantly folded proteins might also be provided by a mechanism of cell competition [183].
(v). Microglia undergo cell competition under conditions of inflammation and polarization [184].

In sum, the c-Myc protein serves as a key player in the regulation of cell competition in different types of tissues. It should be noted that a significant portion of studies on the role of this protein in the regulation of cell competition machinery were carried out in cells of non-brain origin, mainly in epithelial cells, fibroblasts, stem cells, and tumor cells. However, given that c-Myc expression is recorded almost equally in neurons showing the expression of c-Fos [185], we could presumably afford to draw some parallels between cells of neuronal and non-neuronal lineages in terms of their c-Myc-mediated behavior under the conditions of cell competition.

### 4.1. Cell Competition and Neurogenesis

It is believed that the mechanism of cell competition is most intensively manifested during neurogenesis and is a fundamental component of nervous system development. Cell competition plays a role in regulating neural cell numbers, canceling developmental errors or noise, and tissue remodeling processes [186]. The most obvious example of cell competition is neurogenesis in the embryonic brain or in postnatal neurogenic niches. Cell competition may serve as a quality control mechanism in the developing brain [187,188,189], and the same might be true for the limited neurogenesis in the adult brain, where a new learning performance affects the balance of apoptosis and neurogenesis within neurogenic niches and determines learning outcomes [190,191,192]. Thus, one may assume that experience-driven synaptic activity, the establishment of new neuronal or astroglial networks, and the stimulation of neurogenesis could be linked to the activation of cell competition in the brain.

Is there any role for the brain plasticity-activated expression of IEGs in this mechanism? The role of c-Myc in the control of adult neurogenesis and the accumulating data on the contribution of neurogenesis to learning and memory [193] suggests that the c-Myc-driven proliferation of neural stem and progenitor cells, as well as the regulation of the apoptosis of newly formed cells within neurogenic niches might be important for brain plasticity.

The establishment of neuronal ensembles with signs of hyperexcitation (e.g., loci of seizures, the development of glutamate excitotoxicity) may result in the apoptosis and enhanced proliferation of cells in neurogenic niches—SGZ and SVZ [194,195]. Thus, it could stimulate cell competition between newly formed progenitors and immature neuronal cells. At the same time, the phenomenon of Arg3.1/Arc expression in young neurons at the early postmitotic stage in neurogenesis has been observed: these cells are not sensitive to the synaptically induced expression of IEGs, but demonstrate the constitutive expression of Arg3.1/Arc (not other IEGs, e.g., c-Fos, Zif268, or Homer1a) [88]. Thus, it is assumed that young Arg3.1/Arc+ neurons represent a subpopulation with higher viability and a better ability to integrate into the pre-existing neural networks required for memory consolidation [94]. If so, the expression of Arg3.1/Arc at the earliest stages of neurogenesis could mark the subpopulation of “winners” in the SGZ, whose viability is not very affected by the presence of hyperexcited neurons in the hippocampus.

Even the synthesis and degradation of the c-Fos protein is part of cell homeostasis, but its overexpression leads to increased cell proliferation [67]. This might help to explain the function of c-Fos as a regulator of brain cell development: c-Fos staining is a reliable and sensitive biomarker of hippocampal–enthorinal network maturation [77]. c-Fos knockout animals demonstrate numerous developmental abnormalities and growth retardation [196,197]. The expression of ΔFosB positively affected the proliferation of neuronal progenitor cells in a rat model of brain ischemia, thereby contributing to neuroprotection [198]. In many other tissues, the expression of c-Fos is associated with cell proliferation, differentiation, migration, and apoptosis [67]. Since IEGs are the targets for ERK-mediated phosphorylation [79,199] and ERKs are involved in the mechanisms of cell competition in various tissues [200], the phosphorylation of IEGs in brain cells might contribute to cell competition. Indeed, c-Fos, as well as another IEG, c-Myc, may act as intracellular ERK-signaling sensors [79]. During embryonic neurogenesis in mice, the expression of c-Myc in neuronal progenitor cells is required for their proliferation and neuronal differentiation [201]. This activity of c-Myc is not only evident in the embryonic brain: in neurogenic niches of the adult brain, Myc expression drives neurogenesis and oligodendrogenesis [202] via controlling mitochondrial remodeling and cell cycle transition in neural stem cells [203]. However, the results of Wang et al. showed that c-Myc blocked the differentiation of neuronal progenitor cells into neurons [136].

### 4.2. Cell Competition and Brain Metabolism

Another mechanism of IEGs’ contribution to cell competition in the brain might be linked to their regulatory activity on the cellular metabolism. Brain plasticity is always coupled with significant changes in brain cells’ metabolism. One of the first examples regarding this could be considered the difference in the effect of nerve growth factor on the metabolic activity of different neurons [204]. It has been shown that activated neurons that mainly depend on mitochondrial activity for ATP production are needed in the additional influx of glycolytically produced lactate from astrocytes within the tripartite synapses [189]. At the same time, in neurogenic niches, neural stem cell recruitment requires a switch from glycolysis to mitochondrial respiration to allow for the efficient differentiation and migration of newly formed neuronal cells [191].

Numerous metabolic changes have been attributed to the phenomenon of cell competition and the elimination of cells from the population. According to Chambers et al., “winners” should demonstrate elevated protein synthesis and glycolysis, whereas the suppression of mitochondrial oxidative phosphorylation (OXPHOS) results in the apoptosis of “losers” [192]. The inactivation of mitochondrial respiration might be linked to the KNL-dependent phosphorylation of PDH, which decreases the ability of “loser” cells to convert pyruvate into acetyl-CoA [205]. This metabolic reprogramming is switched on in hypoxia-exposed cells using an HIF-1-dependent mechanism [206]. Switching the cell metabolism from OXPHOS to glycolysis requires significant changes in mitochondrial dynamics, notably the induction of excessive mitochondrial fission and fragmentation [207].

In physiological conditions, the activity of GSK3β, whose participation in synaptic plasticity is achieved via insulin receptor signaling, is suppressed, but in neurodegeneration, the progression of cerebral insulin resistance results in abnormally elevated activity of the enzyme in neuronal cells [208]. Cell competition was confirmed to be related to different metabolic activities of interacting cells following an initial study [209], which demonstrated alterations in the competitiveness of cells with disturbed insulin receptor-triggered signaling pathways in flies due to the abnormal activity of the IRS protein. More recent studies suggest that insulin resistance and hyperinsulinemia suppress cell competition in the epithelium, thereby promoting the excessive proliferation of polarity-deficient cells that would normally be eliminated by surrounding wide-type cells [210]. Whether a similar mechanism might be activated in neurodegeneration is an open question.

PDH is a key enzyme linking glycolytic activity and mitochondrial respiration; its phosphorylation by PDK results in the inhibition of pyruvate’s conversion into acetyl-CoA, whereas an abnormally high phosphorylation of PDH is seen in insulin resistance [211]. c-Jun N-terminal kinase (JNK) phosphorylation and its translocation to mitochondria in neurons is associated with PDH phosphorylation, and this mechanism is accelerated during aging [212]. A recent study revealed that the phosphorylation of PDH is inversely correlated with the intensity of potential firing in neurons, and pPDH can be used, together with IEGs (like c-Fos), as negative and positive markers of experience-driven neuronal activity, respectively [213].

**Figure 5:**
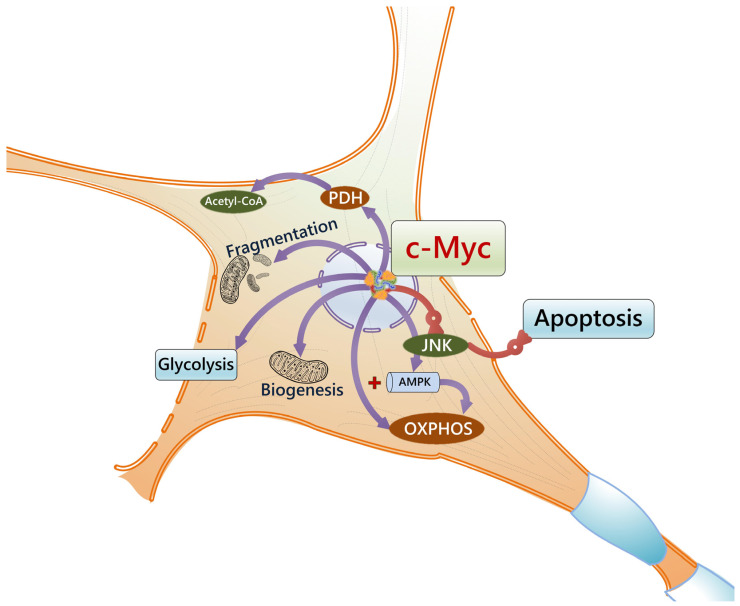
The overexpression of c-Myc results in increased glycolysis, alters mitochondrial morphology (mitochondrial fragmentation), stimulates mitochondrial biogenesis and mitochondrial activity, as well as the PDH-mediated production of acetyl-CoA, and suppresses JNK-mediated apoptosis. c-Myc overexpression leads to the depletion of ATP levels and activation of AMPK. c-Myc and AMPK could work together to maintain a positive balance of ATP and AMP and to allow for effective proliferation through coordinating the efficacy of OXPHOS and glycolysis.

How are such events connected to metabolic alterations in competing cells? In cortical neurons, the JNK-mediated suppression of PDH activity leads to elevated lactate levels and concomitant NAD+ regeneration to support glycolysis [214]. This mechanism is reasonable for actively proliferating cells since the regeneration of NAD+ due to the LDH-mediated conversion of pyruvate into lactate would support glycolytic flux and the biosynthesis of nucleotides and amino acids. Moreover, well-known regulators of cell competition, such as Ras and c-Myc, may positively affect the JNK cascade [215]. However, some authors [205] suggested that loser cells produce lactate to support the viability of the winners. Actually, this mechanism seems to be very close to the Warburg’s effect and reverse Warburg’s effect seen in tumor cells and the tumor microenvironment [216] and to the neuron–astrocyte metabolic coupling, which is important for brain plasticity [217]. The overexpression of c-Myc results (Figure 5) in increased glycolysis and alters mitochondrial morphology due to the elevated mitochondrial fragmentation [218]. However, in some cells, c-Myc may stimulate mitochondrial biogenesis and mitochondrial activity, as well as the PDH-mediated production of acetyl-CoA, and suppress JNK-mediated apoptosis [219,220,221,222]. Thus, mammalian cells with high c-Myc expression should demonstrate preferable mitochondrial generation of ATP.

c-Myc overexpression leads to the depletion of ATP levels and activation of AMPK, which is required for mitochondrial biogenesis and OXPHOS, but their cooperative activity is more complicated: it was proposed that they could work together to maintain a positive balance of ATP and AMP and to allow for effective proliferation through coordinating the efficacy of OXPHOS and glycolysis [223]. In embryonic tissues, the elimination of less fit cells is associated with the inhibition of mTOR signaling in a p53-dependent manner [224]. In non-proliferating tissues in postnatal ontogenesis, the elimination of cells via the cell competition mechanism requires cellular hypertrophy to repair the tissue [225]. For instance, cellular hypertrophy is driven by mTOR machinery in skeletal muscle cells [226], cardiac cells [227], and neurons [228]. Even acute mTOR inhibition may induce insulin resistance and lower glucose utilization in muscle cells [229]. The activity of mTOR is under the negative control of AMPK which detects the levels of ATP and AMP, drives mitochondrial activation, and suppresses glycolysis in cells [230]. Thus, one may suggest that, in the context of cell competition, the “winners” should have higher Myc-driven OXPHOS activity, but the secondary activation of AMPK caused by the excessive production of ROS in mitochondria may stimulate the establishment of the Warburg effect needed for cell survival [223].

mTOR inhibitors may improve the lifespan in vivo and suppress the aging program, presumably acting via mTORC1 (which is regulated mainly by insulin, ATP, phosphatidic acid, and amino acids, and is normally responsible for the activation of glycolysis, the pentose phosphate pathway, the biosynthesis of fatty acids and cholesterol, ribosomal protein synthesis, cell growth, and the suppression of autophagy), whereas the inhibition of mTORC2 (which is normally linked to the insulin and IGF signaling cascade driving cell survival, cytoskeletal remodeling, and cell migration) is related to alterations in glucose and lipid metabolism, as well as to a negative effect on lifespan [231,232,233]. More importantly, mTORC1 provides the negative feedback regulation of IRS via serine phosphorylation, whereas mTORC2 acts as a tyrosine kinase for IR and IGFR, as well as being an inducer of IRS-1 degradation [233]. Thus, under physiological conditions, mTORC1 signaling is important for preventing the excessive action of insulin and mTORC2, but under conditions of insulin resistance, hyperinsulinemia, and obesity, chronic mTORC1 overactivity may further attenuate insulin and mTORC2 signaling and trigger the establishment of circulus vitiosus [234]. It should be noted that the effects of mTOR machinery activation might depend on the cell type, e.g., a low-protein diet suppresses mTORC1 signaling in proliferating cancer cells but increases mTORC1 signaling in tumor-associated non-proliferating macrophages, and as a result, tumor cells are efficiently eliminated from the tissue [235].

The data obtained by Rawat et al. show the protective effects of enhanced c-Fos expression in cortical neurons due to homocysteine toxicity, while its knockdown caused the death of healthy neurons [236]. This suggests that c-Fos is necessary for the survival of neurons and an increase in its expression has a neuroprotective effect [237]. Since c-Fos expression is driven by numerous physiological and pathological stimuli, including neurotransmitters, cytokines, growth factors, stress signals, and proinflammatory molecules, its expression might have different outcomes in different cells: for example, it may stabilize LTP in neurons, respond to noxious, stressful, or proinflammatory signals in glial cells, and cause metabolic changes in various types of cells. The latter effect is confirmed by the finding of an association between the c-Fos protein and the endoplasmic reticulum; it was found to control the lipid metabolism and stimulate the activity of lipid synthesizing enzymes (e.g., CDP-diacylglycerolsynthase-1, phosphatidylinositol-4-kinase type II α) in neuronal cells [49]. In other cell types (e.g., chondrocytes), c-Fos regulates energy production by balancing the pyruvate flux and anaerobic glycolysis and the tricarboxylic acid cycle in response to proinflammatory stimuli [48].

### 4.3. Cell Competition and Synaptic Plasticity

The phenomenon of synaptic competition might be linked to the competitive behavior of different synapses in a neuron because of their intrinsically different strengths or the limited number of plasticity-related products like BDNF, Arc3.1/Arc, PKMζ, PSD proteins, etc., as well as transcription factors promoting their expression [238]. Alterations in these mechanisms might result in the deregulation of LTP and LTD, an excitation-to-inhibition imbalance, and the appearance of neurons with abnormally enhanced excitability, as observed in the initial stages of Alzheimer’s type neurodegeneration [239].

When LTP is almost “saturated”, further learning is impossible [240]; this is controlled by the induction of LTD. Synaptic activity may initiate apoptotic cascades, resulting in the cleavage of ionotropic glutamate–receptor subunits [190], and, presumably, the death of neurons with a weaker ability to support LTP and LTD. Additionally, synapses may have various patterns of activation leading to NMDA receptor signaling and the development of LTP or LTD, and such variability depends on the activity of GSK3α/GSK3β: an active enzyme is required for the induction of LTD [241,242] and structural plasticity of dendritic spines [242]. Moreover, Arg3.1/Arc was identified as a substrate for GSK3β-mediated phosphorylation, which is required for the degradation of Arg3.1/Arc proteins [163]. At the same time, inhibitors of GSK3β in neuroblastoma cells reduce c-Myc expression and promote apoptosis [243].

The intrinsic excitability of neurons is known to be modulated by synaptic activity [244]. Intrinsic plasticity, in turn, depends on the activity of synaptic receptors (NMDAR, mGluR, AMPAR) [245,246,247]. Furthermore, the NMDAR-dependent induction of synaptic plasticity is associated with c-Fos activation [73]. It is also known that NMDAR and mGluR stimulation activates Arg3.1/Arc transcription in neurons (via ERK, PKA, and PKC protein kinases), which is associated with the increased endocytosis of AMPARs [91,92]. Thus, IEGs may be involved in the mechanism of regulation of internal plasticity and the excitability of neurons.

In neurons, only those axons that contain clusters of mTOR respond to the mTOR-dependent signal and transmit the trophic signal back to the presynaptic cell body, causing the response necessary for long-term plasticity [235]. Therefore, it is likely that synaptic plasticity is a possible variant of the competitive behavior of neurons: an increase in the expression of mTOR in axons leads to an increase in the metabolic response of the entire neuron, which is necessary for long-term plasticity. This is confirmed by in silico models for a privileged subgroup of synapses defined by the mTOR cluster in the mechanisms of synaptic plasticity in the establishment of long-term memory [248,249]. Thus, the competitive behavior of activated neurons may be linked to the “competition” between strong and weak synapses driven by the difference in the expression of some IEGs, particularly c-Fos, Arg3.1/Arc, and c-Myc.

In addition to neurochemical signaling and presynaptic stimulation, changes in the level of coherence between pre- and postsynaptic activity (spike-timing-dependent plasticity (STDP)) are involved in the process of LTP and LTD formation [250]. This pathway is considered to be more physiological for the induction of synaptic plasticity [251,252]. It could be hypothesized that IEGs are also involved in this mechanism. Thus, the commonly accepted model of STDP induction suggests that LTP is triggered by an increase in calcium influx through unblocked synaptic NMDARs [251,253]. An in vitro model also found that STDP alters synaptic the strength of electrosensory neurons via NMDARs [254], which is associated with the activation of c-Fos and Arg3.1/Arc [73,91,92].

## 5. Aberrant Arg3.1/Arc, c-Fos, and c-Myc Expression and Cell Competition in Brain Aging and Neurodegenerative Diseases

Aberrant cell competition in either stem cells or mature cells in the tissue is now recognized as a significant contributor to aging [255,256] and neurodegeneration as a type of advanced aging [257]. Aging is a major risk factor for the development of most neurodegenerative diseases and is accompanied by a decline in cognitive function in a significant part of the population. A characteristic sign of such changes is the loss of explicit memory against the background of the preservation of implicit memory. Natural cognitive brain dysfunction, characteristic of older people, has certain similarities with the changes observed in neurodegenerative diseases, but the latter are characterized by the phenomenon of accelerated aging. During the long-term latent stage of neurodegeneration, due to the mechanisms of neuroplasticity, metabolic plasticity, and neural network rearrangements, clinical symptoms of brain damage may be absent for a long time [258].

Aging and chronic neurodegeneration are characterized in the brain by the loss of neuronal maturity, which is associated with changes in the expression profile and excitability of cells [259]. This phenomenon is triggered by the inflammation and hyperexcitation of cells and may even be associated with cell re-entry into the cell cycle. Most likely, the subsequent fate of such cells depends on the extent to which they are able to maintain viability (not so much in the neurogenic niche, but in brain tissue that does not have a microenvironment specialized for stem/progenitor cells) and effectively form connections with other neurons. Another mechanism that is attributed to aging is altered cell competition [256,260].

A growing body of research highlights the interplay between IEGs, such as c-Fos, and cell competition —both of which are crucial for maintaining tissue integrity and cellular fitness during aging. Deciphering these mechanisms will provide new avenues for therapeutic interventions that may not only extend the lifespan but also improve the healthspan, the period of life free from disease [260,261].

During aging, the deregulation of IEGs is linked to both normal aging processes and age-related diseases [262]. For instance, recent studies revealed a role of c-Jun in the regulation of short-range interactions among neurons, resulting in abnormal cell fitness and cell competition in Alzheimer’s type neurodegeneration [263]. In Alzheimer’s disease, increased c-Fos expression was observed in surviving hippocampal neurons, potentially indicating a compensatory mechanism or reflecting the degeneration of affected neurons. Animal models of AD have shown that external stressors, such as exposure to noise, exacerbate neurodegeneration by promoting changes like tau hyperphosphorylation and amyloid-beta accumulation [264]. These pathological processes are associated with elevated c-Fos expression, suggesting that this gene plays a role in the brain’s stress response mechanisms during disease progression [265]. The expression of IEGs and cell competition are interconnected in their roles maintaining tissue and cellular fitness during aging [56,256]. Both mechanisms are involved in the brain’s response to inflammaging, influencing the fate of neurons and other cell types. As c-Fos and other IEGs regulate synaptic plasticity and stress responses, their deregulation can impair cell competition, leading to the accumulation of damaged or senescent cells that contribute to disease progression [56].

In addition, c-Fos expression in response to neuronal injury or environmental stress has been investigated in various models, such as rodents and zebrafish, further emphasizing its role in cell survival, synaptic plasticity, and neurodegenerative diseases [266]. However, as organisms age, the regulation of c-Fos becomes impaired, with reduced expression in certain brain regions [264]. This decline is linked to reduced brain function and may contribute to the increased vulnerability of aged neurons to stress and damage [266].

Elevated c-Fos levels in neurons during neurodegenerative diseases like Alzheimer’s may be linked to a failure in cell competition, where the brain becomes less efficient at eliminating weaker neurons [184]. This decline in cell competition could accelerate cognitive decline by allowing for the accumulation of less fit cells, exacerbating neurodegeneration. Conversely, enhancing cell competition could support the survival of cells with higher fitness and improve tissue integrity, potentially offering therapeutic benefits in age-related diseases [56].

In the immune system, cell competition also plays a role in regulating microglial turnover in the brain. Microglia, the brain’s resident immune cells, maintain themselves through self-renewal, and their survival is influenced by factors like interleukin-34 and the colony-stimulating factor-1 receptor (CSF1RA). Age-related reductions in CSF1RA expression diminish the competitiveness of certain microglia populations, leading to their decline with age. This suggests that modulating cell competition in microglia could help sustain brain health during aging [184].

As organisms age, long-lived cells like neurons and glia also accumulate damage. In Drosophila, neurons and glial cells undergo polyploidization—a process in which cells acquire multiple copies of their genome, which helps protect them from DNA damage and oxidative stress. There is polyploidy in neuronal cells, which occurs due to their re-entry into the cell cycle [267]. Despite the potential protective role of neuronal polyploidy, studies have revealed a link between the resumption of the cell cycle in neurons and neurodegeneration [268]. In addition, exogenous DNA damage and oxidative stress can cause even higher levels of polyploidy in cells that are protected from cell death [268]. Interestingly, a link has been found between activation of the mTOR signaling pathway and signs of polyploidy [269]. If, in postmitotic neurons, c-Myc provokes re-entry into the cell cycle, which causes endoreplication without mitosis and the appearance of polyploid cells in the postnatal brain [270], then the formation of polyploid neurons can be considered an attempt to survive cell competition under conditions of cellular stress. Indeed, the ectopic expression of c-Myc in neurons causes cell cycle re-entry and neurodegeneration [129,271].

Polyploid cells demonstrate increased resistance to damage-induced cell death compared to diploid cells, particularly in brain regions such as the optic lobes. This suggests that polyploidization may serve as an adaptive response to preserve neuronal and glial function during aging [266]. However, polyploidy is a double-edged sword in the nervous system. While it can offer protection against damage, abnormal cell cycle re-entry in neurons has been linked to neurodegeneration. Neurons that re-enter the cell cycle under pathological conditions often die, contributing to diseases like Alzheimer’s [268]. c-Myc has been implicated in this process, as it is upregulated in response to neurotoxic agents, such as amyloid-beta, leading to neurodegeneration through cell cycle re-entry. In Parkinson’s disease, c-Myc deregulation has also been linked to mitochondrial dysfunction and neuronal death, further illustrating its role in neurodegenerative processes [124,272]. An aberrant expression of c-Myc was found in the brain of individuals with AD [273].

The potential role of Arg3.1/Arc in AD is indicated by two key findings: (i) single-nucleotide polymorphisms in the 3′ UTR of Arg3.1/Arc are associated with an increased susceptibility to AD [274]; (ii) Arg3.1/Arc protein levels in the medial frontal cortex are elevated in patients with AD vs. age-matched healthy persons [177]. A study using a transgenic Drosophila model of tauopathy linked ArcD1 accumulation in the nucleus to tau-induced mRNA overexpression and mRNA quality control impairment [275,276]. Arc1 intranuclear accumulation in transgenic flies can suppress GluA1 transcription by increasing the number of PML nuclear bodies, leading to an increase in H4K12 acetylation via Arc1-Tip60 interaction. This Arc-mediated chromatin modification is associated with the increased transcription of anti-inflammatory genes and other IEGs known to be involved in the pathogenesis of AD [277]. With age, especially in patients with AD, a dramatic increase in Arg3.1/Arc interactions with filamin A (FLNA), instead of PSD95, is observed, resulting in a dominant presence of Arg3.1/Arc in FLNA signaling complexes, a critical scaffold protein, modulating a variety of receptors and signaling molecules. This change in binding behavior patterns indicates a regulatory shift as the pathological condition progresses, which may be largely related to synaptic disturbances in AD and aging. An altered FLNA conformation is closely associated with amyloid plaque and tau tangle formation in AD and may also cause Arg3.1/Arc accumulation in FLNA-dependent signaling pathways, including α7nAChR, TLR4, and IR [278,279].

Like c-Fos, in addition to the known control functions of LTP and LTD, Arc regulates some other mechanisms that are important for cellular survival and, possibly, cell competition in the brain tissue. In activated neurons, Arc/Agr3.1 promotes LTP development through regulating dynamic changes in the actin cytoskeleton and the endocytosis of vesicles containing glutamate receptors (AMPAR). In addition, it induces secondary epigenetic changes and the proteolytic degradation of Notch1 (Notch homolog protein 1) or APP, affects the expression of the PSD protein complex, and transfers its own mRNA in Arc-capsid to neighboring cells [3,4,70]. Arg3.1/Arc controls the signaling mechanism initiated by proteolysis of the Notch protein, whose ligands are proteins of the Delta and Jagged families [280]. The Arg3.1/Arc complex with dynamin positively regulates the γ-secretase-mediated cleavage of Notch1 in neurons and leads to an increase in the production of the intracellular fragment—NICD (Notch intracellular domain). It is known that Notch signaling regulates many events in neuroplasticity; notably, the decrease in Notch expression suppresses LTP and increases LTD in the synapses of hippocampal neurons [281]. Thus, it is likely that Notch signaling is involved in the mechanisms of the Arc-mediated modulation of synaptic transmission. APP proteolysis is important for numerous physiological processes (neurogenesis, synapse stabilization, and cerebral angiogenesis), but is altered in the case of Alzheimer’s type neurodegeneration [282]. The products of γ-secretase activity at APP include not only extracellular β-amyloid peptides, but also AICD (APP intracellular domain). The latter, which is associated with the Fe65 adapter protein, is protected from cytosolic degradation and migrates to the cell nucleus to form a complex with histone acetyltransferase Tip60 [283]. Such events are associated with the activation of neurons; the activity-dependent generation of β-amyloid and AICD requires the expression of Arc. Then, Arg3.1/Arc interact with the subunit of γ-secretase, the protein presenilin (PS1), which ensures the localization of γ-secretase in dendritic endosomes for APP proteolysis: in the postsynaptic neuron, APP is internalized as part of the endosomes, where it is processed. The suppression of Arg3.1/Arc’s interaction with γ-secretase prevents the generation of β-amyloid in an activity-dependent mode [284]. Therefore, it is not surprising that, in some animal models of AD, it is possible to detect the Arc-mediated hyperproduction of β-amyloid, which is preceded by the inactivation of GSK3α/β, the activation of Wnt signaling, the accumulation of Arg3.1, and the progression of neuroinflammation [285].

Less is known regarding the role of aberrant Arg3.1/Arc expression in the pathogenesis of Parkinson’s disease (PD). Arg3.1/Arc knockout mice demonstrate dopaminergic dysfunction, similarly to the phenotype of PD [286]. Cellular α-synuclein fate may be determined through Arg3.1/Arc’s protein-regulatory effect on lipid raft function in neuron membranes [164]; this suggests a role of aberrant Arg3.1/Arc expression in PD progression.

Obvious changes in Arg3.1/Arc expression were registered in elderly patients with cognitive deficits. A direct relationship was found between Arg3.1/Arc expression aberrations in the hippocampus and age-related altered cognitive state associated with epigenetic regulation disturbances [287]. However, the intricate relationship between Arc dynamics and cognitive aging remains complex and, at times, elusive [288]. As a member of the IEG family, Arg3.1/Arc is integral to the processes that underpin memory consolidation and synaptic plasticity. As the brain ages, the expression of Arg3.1/Arc and other related IEGs, such as Egr1/Zif268 and Npas4, significantly declines, particularly in the dentate gyrus of the hippocampus—a brain region essential for memory formation, neurogenesis, and spatial navigation [289].

This age-related reduction in IEG expression is closely linked to the cognitive impairments observed in older individuals. It is suggested that these changes might be driven more by alterations in DNA methylation than merely a decrease in neuronal activity. The downregulation of IEGs, including Arg3.1/Arc, is believed to contribute to the deficits in synaptic plasticity seen in aging, potentially leading to delayed spatial memory formation and a marked reduction in adult neurogenesis [290,291]

Despite the clear association between altered Arc expression and cognitive decline, the precise molecular mechanisms by which Arg3.1/Arc contributes to aging-related cognitive impairments are not fully understood. A study of genetic variations in Arg3.1/Arc found only nominal correlations with cognitive performance in middle-aged adults [291]. This suggests that the common genetic variants in Arg3.1/Arc may not significantly account for the variability in cognitive abilities among individuals.

It should also be noted that epigenetic regulation mechanisms play an important role in the regulation of IEG expression. For example, the increased acetylation of histone H4K8 in exon 1 of the CB1R gene causes neonatal neurodegeneration through inhibiting CREB phosphorylation and decreasing Arc expression [292]. HDAC-mediated histone deacetylation was also shown to impair the gene expression of several synaptic plasticity genes, such as brain-derived neurotrophic factor, c-Fos, Egr1, and Arc [293]. At the same time, Subbanna et al. found that the suppression of DNA methylation impairs the activation of extracellular kinase ERK1/2 and decreases Arc expression, as well as causing neurodegeneration in newborn mice and behavioral abnormalities in adult mice [294]. In addition, the role of non-coding RNAs in the epigenetic regulation of IEG expression is evident. As such, increased levels of miR-132/miR-212 and miR-134 are associated with a persistent decrease in the expression of synaptic plasticity genes Arc, c-Fos, and CREB, and are seen in neurocognitive disorders [295].

The synaptic tagging and capture (STC) model, which is fundamental to our understanding of synaptic plasticity, further highlights Arc’s importance. The STC model explains how memories are consolidated through LTP through tagging activated synapses, which subsequently capture the plasticity-related products necessary for transforming short-term memories into long-term ones. Aging disrupts STC-related processes, making it more challenging to form new memories, as the aged hippocampus exhibits altered plasticity, with enhanced LTD and reduced LTP [296]. While substantial evidence supports the critical role of Arg3.1/Arc in cognitive aging, many questions remain regarding the underlying molecular mechanisms. The interplay between Arc and other synaptic plasticity processes continues to be a pivotal area of research, which is crucial for understanding and potentially mitigating age-related memory impairments. Further studies are needed to unravel these complex interactions, which could pave the way for targeted interventions aimed at preserving cognitive function in aging populations.

## 6. Conclusions and Future Prospects

Brain plasticity implies the combination of numerous events linked to short-term and long-term changes in the expression pattern and metabolism of neuronal and non-neuronal cells, collective cell behavior, the excitability of neurons, the development of new young neurons and death of old or damaged ones, and the establishment of multicellular networks at short and long distances from each other within the brain tissue. Some, if not all, of these mechanisms are driven by the coordinated expression of IEGs that encode transcription factors, affecting the expression of a secondary set of genes in activated neurons and thereby serving as markers of neuronal activity, but also acting as multitask cellular proteins involved in the complex regulation of cell metabolism, proliferation, differentiation, competition, and viability.

Taking into consideration the idea of cell competition, which explains many events underlying collective cell behavior within tissues, one may assume that brain plasticity might be directly linked to this phenomenon, partially driven by IEGs in activated cells. In general, the most competitive neurons should have the following characteristics: (i) a balanced LTP and LTD in response to (patho)physiological stimuli; (ii) the correct remodeling of synapses facilitating effective synaptic transmission; (iii) an adaptive mode of cellular metabolism (metabolic reprogramming or metabolic plasticity), particularly in the context of switching between different means of ATP production (glycolysis and mitochondrial OXPHOS) and corresponding changes in mitochondrial dynamics; (iv) the ability to maintain the expression of key regulatory genes, including IEGs, even under stressful conditions (hypoxia, shortage of nutrients, reductive or oxidative stress, accumulation of improperly folded or aggregated proteins, aberrant cell cycle re-entry, inflammation, etc.); (v) the ability to integrate into pre-existing neuronal networks or to establish and maintain new ones; (vii) the ability to escape physiological cell elimination and keep the changes induced by neuronal activity for a long time. In addition, the timely replenishment of a neuronal pool, resulting in the appearance of young, highly excitable, and viable neurons that are able to establish new interneuronal connections, should accompany the mechanisms of brain plasticity.

As we have discussed above, the balanced expression of c-Fos and Arg3.1/Arc in stimulated neurons (e.g., in learning) drives the establishment of LTP and LTD and excitation-to-inhibition balance, supports the establishment of PSD and competitive elimination of weak synapses, enhances the strength of synaptic connections between activated neurons, and prevents the development of hyperexcitation in neuronal cells, which might be destructive to the establishment of engrams. Thus, a lack of this mechanism would result in the appearance of hyperexcited neuron loci with frequent cell cycle re-entry and/or cell death [137], followed by the stimulation of neurogenesis in an ERK-dependent manner [297,298]. Then, c-Myc-mediated pathways could be activated in the neural stem and neuronal progenitor, leading to the adjustment of their metabolism to the energy requirements for cell recruitment, proliferation, and differentiation. At the same time, the expression of Arg3.1/Arc in young, recently generated, immature neurons would support their viability and integration into the pre-existing neuronal circuits [94,299]. As a result, IEGs-driven competition might be important at the synaptic level in experience-activated neurogenesis, as well as in the elimination of aberrantly functioning cells. However, such mechanisms require further experimental assessment and clarification.

In sum, almost all these characteristics, which couple the phenomena of experience-induced brain plasticity and cell competition, might be effectively controlled by IEGs, particularly c-Fos, Arg3.1/Arc, and c-Myc (Table 1). Moreover, IEG-driven changes in neuronal and/or glial cell phenotype might result not only in the establishment of stable and effective cell-to-cell communication, but also in improvements in fitness state to increase the survival of cells that will later contribute to long-term brain plasticity.

The physiological aging and advanced aging of the brain during neurodegeneration are always associated with abnormal brain plasticity and loss of quality control, resulting in the inappropriate elimination of damaged/suboptimal cells, and are characterized by the deregulated expression of IEGs. Therefore, deciphering the molecular mechanisms underlying the IEGs-mediated control of cell competition in the brain might be beneficial for the development of novel strategies to suppress progressive neuronal loss and cognitive decline in aging and neurodegeneration.

## Figures and Tables

**Table 1:** Characteristics that are under the control of IEG-encoded proteins in neuronal cells.

Parameters	Confirmed Regulatory Activity of IEG-Encoded Proteins in Neuronal Cells
Balance of LTP and LTD, effective synaptic remodeling	c-Fos: LTP stabilization via CREB/c-Fos system Arg3.1/Arc: LTP and LTD balanced via endocytosis of AMPARs; interaction with PSD95
Metabolic reprogramming in activated cells	c-Fos: stimulation of neuronal lipid synthesis Arg3.1/Arc: activation of GSK3β c-Myc: regulation of glycolysis, mitochondrial dynamics (fragmentation and biogenesis), mitochondrial OXPHOS, AMPK, mTOR activity, and glucose utilization
Key genes expression in cellular stress	c-Fos, Arg3.1/Arc, c-Myc: presence of internal ribosome entry sites (IRES) in the gene enabling cap-independent transcription in stressful conditions; positive regulation of the expression of numerous genes
Cell integration into multicellular networks	c-Fos: stimulation of neuronal networks’ formation (e.g., engram cells) Arg3.1/Arc: intercellular propagation via virus-like capsids
Viability of activated cells vs. non-activated or damaged cells	c-Fos: support of mature neuronal survival Arg3.1/Arc: supports newborn neuronal survival, higher expression under conditions of chaperone-inducing stress resulting in reduced survival of affected cells (elimination of damaged cells) c-Myc: support of cell fitness, regulation of apoptosis, induction of cell cycle re-entry and neuronal loss, regulation of AMPK- and mTOR-coupled signaling pathways
Effective replenishment of cells	c-Fos: stimulation of embryonic neurogenesis, induction of lipids’ biosynthesis for membrane biogenesis, coordination of ERK signaling Arg3.1/Arc: stimulation of BDNF expression, regulation of Notch and APP signaling, stimulation of postnatal neurogenesis c-Myc: stimulation of neurogenesis, NSCs recruitment and proliferation, suppression of NPCs’ differentiation

## Data Availability

Not applicable.

## Abbreviations

α7nAChR	Alpha-7 nicotinic receptor
AD	Alzheimer’s disease
AICD	intracellular domain of amyloid precursor protein
AMPAR	alpha-amino-3-hydroxy-5-methyl-4-isoxazolepropionic acid-type glutamate receptor
AP-1	transcription factor activator protein 1
APP	Amyloid Precursor Protein
Arc	activity-regulated cytoskeleton-associated protein
CREB	CRE-binding protein
csf1ra	colony-stimulating factor-1 receptor
ERK	extracellular regulated kinase
FLNA	filamin A
FMRP	fragile X mental retardation
GluA1	glutamate ionotropic receptor
GSK3	glycogen synthase kinase 3
IEGs	immediate early genes
IRES	Internal Ribosome Entry Site
LTD	long-term depression
LTP	long-term potentiation
mAChR	muscarinic acetylcholine receptor
mGluR	metabotropic glutamate receptor
NICD	Notch intracellular domain
NMDAR	N-methyl-D-aspartate receptor
Notch1	Notch homologue protein
PICK1	protein interacting with C-kinase 1
PKA	protein kinase A activated by cyclic adenosine monophosphate
PKC	protein kinase C activated by diacylglycerol
PML	promyelocytic leukemia protein
PS1	presenilin 1
PSD	postsynaptic density
RNA	ribonucleic acid
SNARE	soluble NSF attachment receptor
Tip60	60 kDa Tat-interactive protein
TCF	ternary complex factor
TLR	toll-like receptor
TrkB	tyrosine kinase B.
TRIM5α	Rhesus protein serving as a restriction factor which affects capsids’ disassembly

## References

[1] Semyanov A., Verkhratsky A. (2021). Astrocytic processes: From tripartite synapses to the active milieu. Trends Neurosci..

[2] Fields R.D. (2020). The Brain Learns in Unexpected Ways: Neuroscientists have discovered a set of unfamiliar cellular mechanisms for making fresh memories. Sci. Am..

[3] Fila M., Diaz L., Szczepanska J., Pawlowska E., Blasiak J. (2021). mRNA trafficking in the nervous system: A key mechanism of the involvement of activity-regulated cytoskeleton-associated protein (Arc) in synaptic plasticity. Neural Plast..

[4] Hantak M.P., Einstein J., Kearns R.B., Shepherd J.D. (2021). Intercellular Communication in the Nervous System Goes Viral. Trends Neurosci..

[5] Lanahan A., Worley P. (1998). Immediate-early genes and synaptic function. Neurobiol. Learn. Mem..

[6] Cortés-Mendoza J., León-Guerrero S.D., Pedraza-Alva G., Pérez-Martínez L. (2013). Shaping synaptic plasticity: The role of activity-mediated epigenetic regulation on gene transcription. Int. J. Dev. Neurosci..

[7] Pérez-Cadahía B., Drobic B., Davie J.R. (2011). Activation and function of immediate-early genes in the nervous system. Biochem. Cell Biol..

[8] Okuno H. (2011). Regulation and function of immediate-early genes in the brain: Beyond neuronal activity markers. Neurosci. Res..

[9] Das S., Lituma P.J., Castillo P.E., Singer R.H. (2023). Maintenance of a short-lived protein required for long-term memory involves cycles of transcription and local translation. Neuron.

[10] Healy S., Khan P., Davie J.R. (2013). Immediate early response genes and cell transformation. Pharmacol. Ther..

[11] Dutcher S.A., Underwood B.D., Walker P.D., Diaz F.G., Michael D.B. (1999). Patterns of immediate early gene mRNA expression following rodent and human traumatic brain injury. Neurol. Res..

[12] Steward O., Schuman E.M. (2001). Protein synthesis at synaptic sites on dendrites. Annu. Rev. Neurosci..

[13] Stacho M., Manahan-Vaughan D. (2022). The intriguing contribution of hippocampal long-term depression to spatial learning and long-term memory. Front. Behav. Neurosci..

[14] Li X.H., Miao H.H. (2019). NMDA receptor dependent long-term potentiation in chronic pain. Neurochem. Res..

[15] Proskura A.L., Malahin I.A., Turnaev I.I., Suslov V.V., Zapara T.A., Ratushnyak A.S. (2013). Intermolecular interactions in neuron functional systems. Vavilov J. Genet. Breed..

[16] Franchini L., Stanic J., Ponzoni L. (2019). Linking NMDA receptor synaptic retention to synaptic plasticity and cognition. Science.

[17] Wang H., Ardiles A.O., Yang S., Tran T., Posada-Duque R. (2016). Metabotropic glutamate receptors induce a form of LTP controlled by translation and Arc signaling in the hippocampus. J. Neurosci..

[18] Sumi T., Harada K. (2020). Mechanism underlying hippocampal long-term potentiation and depression based on competition between endocytosis and exocytosis of AMPA receptors. Sci. Rep..

[19] Campillos M., Doerks T., Shah P.K., Bork P. (2006). Computational characterization of multiple Gag-like human proteins. Trends Genet..

[20] Lüscher C., Malenka R.C. (2012). NMDA Receptor-Dependent Long-Term Potentiation and Long-Term Depression (LTP/LTD). Cold Spring Harb. Perspect. Biol..

[21] Vinci M., Costanza C., Rando R.G., Treccarichi S., Saccone S., Carotenuto M., Roccella M., Calì F., Elia M., Vetri L. (2023). STXBP6 Gene Mutation: A New Form of SNAREopathy Leads to Developmental Epileptic Encephalopathy. Int. J. Mol. Sci..

[22] Praschberger R., Jacquemyn J., Verstreken P. (2021). Molecule-to-Circuit Disease Mechanisms of a Synaptic SNAREopathy. Neuron.

[23] Soler J., Fañanás L., Parellada M., Krebs M.O., Rouleau G.A. (2018). Genetic variability in scaffolding proteins and risk for schizophrenia and autism-spectrum disorders: A systematic review. J. Psychiatry Neurosci..

[24] Kuriu T., Inoue A., Bito H., Sobue K., Okabe S. (2006). Differential control of postsynaptic density scaffolds via actin-dependent and -independent mechanisms. J. Neurosci..

[25] Star E., Kwiatkowski D., Murthy V. (2002). Rapid turnover of actin in dendritic spines and its regulation by activity. Nat. Neurosci..

[26] Minatohara K., Akiyoshi M., Okuno H. (2016). Role of Immediate-Early Genes in Synaptic Plasticity and Neuronal Ensembles Underlying the Memory Trace. Front. Mol. Neurosci..

[27] Miyashita T., Kubik S., Haghighi N., Steward O., Guzowski J.F. (2009). Rapid activation of plasticity-associated gene transcription in hippocampal neurons provides a mechanism for encoding of one-trial experience. J. Neurosci..

[28] Dragunow M., Abraham W.C., Goulding M., Mason S.E., Robertson H.A., Faull R.L. (1989). Long-term potentiation and the induction of c-fos mRNA and proteins in the dentate gyrus of unanesthetized rats. Neurosci. Lett..

[29] Kemp A., Tischmeyer W., Manahan-Vaughan D. (2013). Learning-facilitated long-term depression requires activation of the immediate early gene, c-fos, and is transcription dependent. Behav. Brain Res..

[30] Denny C.A., Kheirbek M.A., Alba E.A., Tanaka K.F., Brachman R.A. (2014). Hippocampal memory traces are differentially modulated by experience, time, and adult. Neuron.

[31] Loebrich S., Nedivi E. (2009). The function of activity-regulated genes in the nervous system. Physiol. Rev..

[32] Penke Z., Morice E., Veyrac A., Gros A., Chagneau C., LeBlanc P., Samson N., Baumgärtel K., Mansuy I.M., Davis S. (2014). Zif268/Egr1 gain of function facilitates hippocampal synaptic plasticity and long-term spatial recognition memory. Philos. Trans. R. Soc. B Biol. Sci..

[33] Baghel M.S., Singh B., Patro N., Khanna V.K., Patro I.K., Thakur M.K. (2019). Poly (I:C) Exposure in Early Life Alters Methylation of DNA and Acetylation of Histone at Synaptic Plasticity Gene Promoter in Developing Rat Brain Leading to Memory Impairment. Ann. Neurosci..

[34] Pollina E.A., Gilliam D.T., Landau A.T., Lin C., Pajarillo N., Davis C.P., Harmin D.A., Yap E., Vogel I.R., Griffith E.C. (2023). A NPAS4–NuA4 complex couples synaptic activity to DNA repair. Nature.

[35] Kim S., Park D., Lee D., Hong S., Yang E., Jeon J., Mori T., Kim H., Kim S., Tabuchi K. (2019). IQSEC3 Mediates Npas4-dependent GABAergic Synaptic Transmission and Depressive-like Behavior. bioRxiv.

[36] James A.B., Conway A., Morris B.J. (2006). Regulation of the Neuronal Proteasome by Zif268 (Egr1). J. Neurosci..

[37] Clark P.J., Bhattacharya T.K., Miller D.S., Rhodes J.S. (2011). Induction of c-Fos, Zif268, and Arc from acute bouts of voluntary wheel running in new and pre-existing adult mouse hippocampal granule neurons. Neuroscience.

[38] Ingi T., Krumins A.M., Chidiac P., Brothers G.M., Chung S., Snow B.E., Barnes C.A., Lanahan A.A., Siderovski D.P., Ross E.M. (1998). Dynamic Regulation of RGS2 Suggests a Novel Mechanism in G-Protein Signaling and Neuronal Plasticity. J. Neurosci..

[39] Tao-Cheng J., Thein S., Yang Y., Reese T.S., Gallant P.E. (2014). Homer is concentrated at the postsynaptic density and does not redistribute after acute synaptic stimulation. Neuroscience.

[40] Yoshii A., Constantine-Paton M. (2007). BDNF induces transport of PSD-95 to dendrites through PI3K-AKT signaling after NMDA receptor activation. Nat. Neurosci..

[41] Mizui T., Ishikawa Y., Kumanogoh H., Lume M., Matsumoto T., Hara T., Yamawaki S., Takahashi M., Shiosaka S., Itami S. (2015). BDNF pro-peptide actions facilitate hippocampal LTD and are altered by the common BDNF polymorphism Val66Met. Proc. Natl. Acad. Sci. USA.

[42] Nambu M.F., Lin Y., Reuschenbach J., Tanaka K.Z. (2022). What does engram encode?: Heterogeneous memory engrams for different aspects of experience. Curr. Opin. Neurobiol..

[43] Zhu X.O., Brown M.W., McCabe B.J., Aggleton J.P. (1995). Effects of the novelty or familiarity of visual stimuli on the expression of the immediate early gene c-fos in rat brain. Neuroscience.

[44] Mehta K., Yentsch H., Lee J., Yook Y., Lee K.Y., Gao T.T., Tsai N.P., Zhang K. (2024). Phosphatidylinositol-3-phosphate mediates Arc capsid secretion through the multivesicular body pathway. Proc. Natl. Acad. Sci. USA.

[45] Zinin N., Adameyko I., Wilhelm M., Fritz N., Uhlén P., Ernfors P., Henriksson M.A. (2014). MYC proteins promote neuronal differentiation by controlling the mode of progenitor cell division. EMBO Rep..

[46] West A.E., Greenberg M.E. (2011). Neuronal Activity-Regulated Gene Transcription in Synapse Development and Cognitive Function. Cold Spring Harb. Perspect. Biol..

[47] Kawashima T., Okuno H., Bito H. (2014). A new era for functional labeling of neurons: Activity-dependent promoters have come of age. Front. Neural Circuits.

[48] Matsuoka K., Bakiri L., Bilban M., Toegel S., Haschemi A., Yuan H., Kasper M., Windhager R., Wagner E.F. (2023). Metabolic rewiring controlled by c-Fos governs cartilage integrity in osteoarthritis. Ann. Rheum. Dis..

[49] Rodríguez-Berdini L., Caputto B.L. (2019). Lipid Metabolism in Neurons: A Brief Story of a Novel c-Fos-Dependent Mechanism for the Regulation of Their Synthesis. Front. Cell Neurosci..

[50] Ryan M.M., Mason-Parker S.E., Tate W.P., Abraham W.C., Williams J.M. (2011). Rapidly induced gene networks following induction of long-term potentiation at perforant path synapses in vivo. Hippocampus.

[51] Mahringer D., Petersen A.V., Fiser A., Okuno H., Bito H., Perrier J., Keller G.B. (2019). Expression of c-Fos and Arc in hippocampal region CA1 marks neurons that exhibit learning-related activity changes. bioRxiv.

[52] Qiao Q., Wu C., Ma L., Zhang H., Li M., Wu X., Gan W. (2022). Motor learning-induced new dendritic spines are preferentially involved in the learned task than existing spines. Cell Rep..

[53] Heo S., Kang T., Bygrave A.M., Larsen M.R., Huganir R.L. (2023). Experience-Induced Remodeling of the Hippocampal Post-synaptic Proteome and Phosphoproteome. Mol. Cell Proteom..

[54] Léveillé F., Papadia S., Fricker M., Bell K.F.S., Soriano F.X., Martel M., Puddifoot C., Habel M., Wyllie D.J., Ikonomidou C. (2010). Suppression of the Intrinsic Apoptosis Pathway by Synaptic Activity. J. Neurosci..

[55] Györffy B.A., Kun J., Török G., Kardos J. (2018). Local apoptotic-like mechanisms underlie complement-mediated synaptic pruning. Proc. Natl. Acad. Sci. USA.

[56] Costa-Rodrigues C., Couceiro J., Moreno E. (2021). Cell competition from development to neurodegeneration. Dis. Model. Mech..

[57] Leuner B., Mendolia-Loffredo S., Kozorovitskiy Y., Samburg D., Gould E., Shors T.J. (2004). Learning Enhances the Survival of New Neurons beyond the Time when the Hippocampus Is Required for Memory. J. Neurosci..

[58] Jeong Y., Cho H., Kim M., Oh J., Kang M.S., Yoo M., Lee H., Han J. (2021). Synaptic plasticity-dependent competition rule influences memory formation. Nat. Commun..

[59] Morgan J.I., Curran T. (1989). Stimulus-transcription coupling in neurons: Role of cellular immediate-early genes. Trends Neurosci..

[60] The Human Protein Atlas FOS Protein Expression Summary. https://www.proteinatlas.org/ENSG00000170345-FOS.

[61] Miyashita T., Kikuchi E., Horiuchi J., Saito M. (2018). Long-Term Memory Engram Cells Are Established by c-Fos/CREB Transcriptional Cycling. Cell Rep..

[62] Bito H., Deisseroth K., Tsien R.W. (1997). Ca^2+^-dependent regulation in neuronal gene expression. Curr. Opin. Neurobiol..

[63] Sheng H.Z., Fields R.D., Nelson P.G. (1993). Specific regulation of immediate early genes by patterned neuronal activity. J. Neurosci. Res..

[64] Velazquez F.N., Caputto B.L., Boussin F.D. (2015). c-Fos importance for brain development. Aging.

[65] Bussolino D.F., Guido M.E., Gil G.A., Borioli G.A., Renner M.L., Grabois V.R., Conde C.B., Caputto B.L. (2001). c-Fos associates with the endoplasmic reticulum and activates phospholipid metabolism. FASEB J..

[66] Kutz S.M., Providence K.M., Higgins P.J. (2001). Antisense targeting of c-fos transcripts inhibits serum- and TGF-beta 1-stimulated PAI-1 gene expression and directed motility in renal epithelial cells. Cell Motil. Cytoskelet..

[67] Lara Aparicio S.Y., Laureani Fierro Á.J., Aranda Abreu G.E., Toledo Cárdenas R., García Hernández L.I., Coria Ávila G.A., Rojas Durán F., Aguilar M.E.H., Manzo Denes J., Chi-Castañeda L.D. (2022). Current Opinion on the Use of c-Fos in Neuroscience. NeuroSci.

[68] Gandolfi D., Cerri S., Mapelli J., Polimeni M., Tritto S., Fuzzati-Armentero M.T., Bigiani A., Blandini F., Mapelli L., D’Angelo E. (2017). Activation of the CREB/c-Fos Pathway during Long-Term Synaptic Plasticity in the Cerebellum Granular Layer. Front. Cell Neurosci..

[69] Lisachev P.D., Shtark M.B. (2018). Long-Term Potentiation-Associated Gene Expression: Involvement of the Tumour Protein p53. The Hippocampus-Plasticity and Functions.

[70] Dragunow M., Faull R. (1989). The use of c-Fos as a metabolic marker in neuronal pathway tracing. J. Neurosci. Methods.

[71] Nakagami Y., Watakabe A., Yamamori T. (2013). Monocular inhibition reveals temporal and spatial changes in gene expression in the primary visual cortex of marmoset. Front. Neural Circuits.

[72] Benn A., Barker G.R., Stuart S.A., Roloff E.V., Teschemacher A.G., Warburton E.C., Robinson E.S. (2016). Optogenetic Stimulation of Prefrontal Glutamatergic Neurons Enhances Recognition Memory. J. Neurosci..

[73] Katche C., Bekinschtein P., Slipczuk L., Medina J.H. (2009). Delayed wave of c-Fos expression in the dorsal hippocampus involved specifically in persistence of long-term memory storage. Proc. Natl. Acad. Sci. USA.

[74] Haghparast A., Taslimi Z., Ramin M., Azizi P., Khodagholi F., Hassanpour-Ezatti M. (2011). Changes in phosphorylation of CREB, ERK, and c-fos induction in rat ventral tegmental area, hippocampus and prefrontal cortex after conditioned place preference induced by chemical stimulation of lateral hypothalamus. Behav. Brain Res..

[75] Nestler E.J. (2004). Molecular mechanisms of drug addiction. Neuropharmacology.

[76] Cunningham J.T., Mifflin S.W., Gould G.G., Frazer A. (2008). Induction of c-Fos and ΔFosB Immunoreactivity in Rat Brain by Vagal Nerve Stimulation. Neuropsychopharmacology.

[77] Pompeiano M., Colonnese M.T. (2023). cFos as a biomarker of activity maturation in the hippocampal formation. Neural Dev..

[78] Cruz-Mendoza F., Jauregui-Huerta F., Aguilar-Delgadillo A., García-Estrada J., Luquin S. (2022). Immediate Early Gene c-Fos in the Brain: Focus on Glial Cells. Brain Sci..

[79] Murphy L.O., MacKeigan J.P., Blenis J. (2004). A network of immediate early gene products propagates subtle differences in mitogen-activated protein kinase signal amplitude and duration. Mol. Cell Biol..

[80] Zhang J., Zhang D., McQuade J.S., Behbehani M., Tsien J.Z., Xu M. (2002). c-Fos regulates neuronal excitability and survival. Nat. Genet..

[81] Flavell S.W., Greenberg M.E. (2008). Signaling mechanisms linking neuronal activity to gene expression and plasticity of the nervous system. Annu. Rev. Neurosci..

[82] Yelhekar T.D., Meng M., Doupe J., Lin Y. (2024). All IEGs Are Not Created Equal-Molecular Sorting Within the Memory Engram. Adv. Neurobiol..

[83] Fleischmann A., Hvalby O., Jensen V., Strekalova T., Zacher C., Layer L.E., Kvello A., Reschke M., Spanagel R., Sprengel R. (2003). Impaired long-term memory and NR2A-type NMDA receptor-dependent synaptic plasticity in mice lacking c-Fos in the CNS. J. Neurosci..

[84] Lyford G.L., Yamagata K., Kaufmann W.E., Barnes C.A., Sanders L.K. (1995). Arc, a growth factor and activity-regulated gene, encodes a novel cytoskeleton-associated protein that is enriched in neuronal dendrites. Neuron.

[85] The Human Protein Atlas ARC Protein Expression Summary. https://www.proteinatlas.org/ENSG00000198576-ARC.

[86] Epstein I., Finkbeiner S. (2018). The Arc of cognition: Signaling cascades regulating Arc and implications for cognitive function and disease. Semin. Cell Dev. Biol..

[87] Zhang W., Wu J., Ward M.D., Yang S., Chuang Y.A., Xiao M. (2015). Structural basis of arc binding to synaptic proteins: Implications for cognitive disease. Neuron.

[88] Zhang H., Bramham C.R. (2021). Arc/Arg3.1 function in long-term synaptic plasticity: Emerging mechanisms and unresolved issues. Eur. J. Neurosci..

[89] Steward O., Worley P.F. (2001). Selective Targeting of Newly Synthesized Arc mRNA to Active Synapses Requires NMDA Receptor Activation. Neuron.

[90] Guzowski J.F., McNaughton B.L., Barnes C.A., Worley P.F. (1999). Environment-specific expression of the immediate-early gene Arc in hippocampal neuronal ensembles. Nat. Neurosci..

[91] Farris S., Lewandowski G., Cox C.D., Steward O. (2014). Selective localization of arc mRNA in dendrites involves activity and translation-dependent mRNA degradation. J. Neurosci..

[92] Steward O., Farris S., Pirbhoy P.S., Darnell J., Van Driesche S.J. (2015). Localization and local translation of Arc/Arg3.1 mRNA at synapses: Some observations and paradoxes. Front. Mol. Neurosci..

[93] Zhang W., Chuang Y.A., Na Y., Ye Z., Yang L. (2019). Arc oligomerization is regulated by CaMKII phosphorylation of the GAG domain: An essential mechanism for plasticity and memory formation. Mol. Cell.

[94] Bramham C.R., Alme M.N., Bittins M., Kuipers S.D., Nair R.R. (2010). The Arc of synaptic memory. Exp. Brain Res..

[95] Patterson M.A., Szatmari E.M., Yasuda R. (2010). AMPA receptors are exocytosed in stimulated spines and adjacent dendrites in a Ras-ERK-dependent manner during long-term potentiation. Proc. Natl. Acad. Sci. USA.

[96] Peebles C.L., Yoo J., Thwin M.T., Palop J.J., Noebels J.L., Finkbeiner S. (2010). Arc regulates spine morphology and maintains network stability in vivo. Proc. Natl. Acad. Sci. USA.

[97] Albanesi J.P., Barylko B., DeMartino G.N., Jameson D.M. (2020). Palmitoylated proteins in dendritic spine remodeling. Front. Synaptic Neurosci..

[98] Jakkamsetti V., Tsai N.P., Gross C., Molinaro G., Collins K.A. (2013). Experience-induced Arc/Arg3.1 primes CA1 pyramidal neurons for metabotropic glutamate receptor-dependent long-term synaptic depression. Neuron.

[99] Jenks K.R., Tsimring K., Ip J.P., Zepeda J.C., Sur M. (2021). Heterosynaptic plasticity and the experience-dependent refinement of developing neuronal circuits. Front. Neural Circuits.

[100] Gong W.K., Ni J., Yu L.F., Wang L., Huang Z.L. (2020). Temporal dynamics of Arc/Arg3.1 expression in the dorsal striatum during acquisition and consolidation of a motor skill in mice. Neurobiol. Learn. Mem..

[101] Messaoudi E., Kanhema T., Soulé J., Tiron A., Dagyte G., DaSilva B. (2007). Sustained Arc/Arg3.1 synthesis controls long-term potentiation consolidation through regulation of local actin polymerization in the dentate gyrus in vivo. J. Neurosci..

[102] DaSilva L.L., Wall M.J., Almeida P.L., Wauters S.C., Januário Y.C., Müller J., Corrêa S.A. (2016). Activity-Regulated Cytoskeleton-Associated Protein Controls AMPAR Endocytosis through a Direct Interaction with Clathrin-Adaptor Protein. eNeuro.

[103] Wall M.J., Corrêa S.A. (2018). The mechanistic link between Arc/Arg3.1 expression and AMPA receptor endocytosis. Semin. Cell Dev. Biol..

[104] Hallin E.I., Bramham C.R., Kursula P. (2021). Structural properties and peptide ligand binding of the capsid homology domains of human Arc. Biochem. Biophys. Rep..

[105] Wall M.J., Collins D.R., Chery S.L., Allen Z.D., Pastuzyn E.D., George A.J., Nikolova V.D., Moy S.S., Philpot B.D., Shepherd J.D. (2018). The Temporal Dynamics of Arc Expression Regulate Cognitive Flexibility. Neuron.

[106] Kyrke-Smith M., Volk L.J., Cooke S.F., Bear M.F., Huganir R.L., Shepherd J.D. (2021). The immediate early gene Arc is not required for hippocampal long-term potentiation. J. Neurosci..

[107] Haley M., Bertrand J., Anderson V.T., Fuad M., Frenguelli B.G., Corrêa S.A.L., Wall M.J. (2023). Arc expression regulates long-term potentiation magnitude and metaplasticity in area CA1 of the hippocampus in ArcKR mice. Eur. J. Neurosci..

[108] Wee C.L., Teo S., Oey N.E., Wright G.D., Van Dongen H.M., Van Dongen A.M. (2014). Nuclear Arc interacts with the histone acetyltransferase Tip60 to modify H4K12 acetylation. eNeuro.

[109] Urban I., Kerimoglu C., Sakib M.S., Wang H., Benito E. (2019). TIP60/KAT5 is required for neuronal viability in hippocampal CA1. Sci. Rep..

[110] Shepherd J.D., Rumbaugh G., Wu J., Chowdhury S., Plath N., Kuhl D., Huganir R.L., Worley P.F. (2006). Arc/Arg3.1 mediates homeostatic synaptic scaling of AMPA receptors. Neuron.

[111] Bloomer W.A., Van Dongen H.M., Van Dongen A.M. (2007). Activity-regulated cytoskeleton-associated protein Arc/Arg3.1 binds to spectrin and associates with nuclear promyelocytic leukemia (PML) bodies. Brain Res..

[112] Miller D.M., Thomas S.D., Islam A., Muench D., Sedoris K. (2012). c-Myc and Cancer Metabolism. Clin. Cancer Res..

[113] Conacci-Sorrell M., McFerrin L., Eisenman R.N. (2014). An overview of MYC and its interactome. Cold Spring Harb. Perspect. Med..

[114] Stine Z.E., Walton Z.E., Altman B.J., Hsieh A.L., Dang C.V. (2015). MYC, metabolism, and cancer. Cancer Discov..

[115] Lancho O., Herranz D. (2018). The MYC Enhancer-ome: Long-range transcriptional regulation of MYC in cancer. Trends Cancer.

[116] Lee C.V.D. (1999). c-Myc target genes involved in cell growth, apoptosis, and metabolism. Mol. Cell Biol..

[117] Kokai E., Voss F., Fleischer F. (2009). Myc regulates embryonic vascular permeability and remodeling. Circ. Res..

[118] Marinkovic D., Marinkovic T. (2021). The new role for an old guy: MYC as an immunoplayer. J. Cell Physiol..

[119] The Human Protein Atlas MYC Protein Expression Summary. https://www.proteinatlas.org/ENSG00000136997-MYC.

[120] Kress T.R., Sabo A., Amati B. (2015). MYC: Connecting selective transcriptional control to global RNA production. Nat. Rev. Cancer.

[121] Guo J., Li T., Schipper J. (2014). Sequence specificity incompletely defines the genome-wide occupancy of Myc. Genome Biol..

[122] Von Der Lehr N., Johansson S., Wu S. (2003). The F-box protein Skp2 participates in c-Myc proteosomal degradation and acts as a cofactor for c-Myc-regulated transcription. Mol. Cell.

[123] MacPherson-Hawthorne K., Sears R.C. (2024). Hold the MYCrophone: MYC Invades Enhancers to Control Cancer-Type Gene Programs. Cancer Res..

[124] Levine M., Cattoglio C., Tjian R. (2014). Looping back to leap forward: Transcription enters a new era. Cell.

[125] Schinelli S., Zanassi P., Paolillo M., Wang H., Feliciello A., Gallo V. (2001). Stimulation of endothelin B receptors in astrocytes induces cAMP response element-binding protein phosphorylation and c-fos expression via multiple mitogen-activated protein kinase signaling pathways. J. Neurosci..

[126] Yoshii A., Constantine-Paton M. (2014). Postsynaptic localization of PSD-95 is regulated by all three pathways downstream of TrkB signaling. Front. Mol. Neurosci..

[127] Wey A., Knoepfler P.S. (2010). C-myc and N-myc in the developing brain. Genes. Cancer.

[128] Li H.L., Dong L.L., Jin M.J., Li Q.Y., Wang X., Jia M.Q., Song J., Zhang S.Y., Yuan S. (2023). A Review of the Regulatory Mechanisms of N-Myc on Cell Cycle. Molecules.

[129] Lee H.P., Kudo W., Zhu X., Smith M.A., Lee H.G. (2011). Early induction of c-Myc is associated with neuronal cell death. Neurosci. Lett..

[130] Marinkovic T., Marinkovic D. (2021). Obscure Involvement of MYC in Neurodegenerative Diseases and Neuronal Repair. Mol. Neurobiol..

[131] Ferrer I., Blanco R., Carmona M., Puig B. (2001). Phosphorylated c-MYC expression in Alzheimer disease, Pick’s disease, progressive supranuclear palsy and corticobasal degeneration. Neuropathol. Appl. Neurobiol..

[132] Tedeschi A., Omura T., Costigan M. (2017). CNS repair and axon regeneration: Using genetic variation to determine mechanisms. Exp. Neurol..

[133] Ma J.J., Ju X., Xu R.J. (2019). Telomerase reverse transcriptase and p53 regulate mammalian peripheral nervous system and CNS axon regeneration downstream of c-Myc. J. Neurosci..

[134] Cai C., Hu X., Dai P., Zhang T., Jiang M., Wang L., Hua W., Fan Y., Han X.X., Gao Z. (2021). c-Myc regulates neural stem cell quiescence and activation by coordinating the cell cycle and mitochondrial remodeling. Signal Transduct. Target. Ther..

[135] Chen P., Chang W.W., Lai Y., Wu C.W. (2009). c-Myc regulates the coordinated transcription of brain disease-related PDCD10-SERPINI1 bidirectional gene pair. Mol. Cell Neurosci..

[136] Wang X.L., Ma Y.X., Xu R.J., Ma J.J., Zhang H.C., Qi S.B., Xu J.H., Qin X.Z., Zhang H.N., Liu C.M. (2020). c-Myc controls the fate of neural progenitor cells during cerebral cortex development. J. Cell Physiol..

[137] Koseoglu M.M., Norambuena A., Sharlow E.R., Lazo J.S., Bloom G.S. (2019). Aberrant Neuronal Cell Cycle Re-Entry: The Pathological Confluence of Alzheimer’s Disease and Brain Insulin Resistance, and Its Relation to Cancer. J. Alzheimer’s Dis..

[138] Ferrer I., Blanco R. (2000). N-myc and c-myc expression in Alzheimer disease, Huntington disease and Parkinson disease. Brain Res. Mol. Brain Res..

[139] Shin H.Y., Kwon M.J., Lee E.M., Kim K., Oh Y.J., Kim H.S., Hwang D.H., Kim B.G. (2021). Role of Myc Proto-Oncogene as a Transcriptional Hub to Regulate the Expression of Regeneration-Associated Genes following Preconditioning Peripheral Nerve Injury. J. Neurosci..

[140] Refaeli R., Kreisel T., Groysman M., Adamsky A., Goshen I. (2023). Engram stability and maturation during systems consolidation. Proc. Natl. Acad. Sci. USA.

[141] Pettit N.L., Yap E.L., Greenberg M.E. (2022). Fos ensembles encode and shape stable spatial maps in the hippocampus. Nature.

[142] Ghandour K., Ohkawa N., Fung C.C.A. (2019). Orchestrated ensemble activities constitute a hippocampal memory engram. Nat. Commun..

[143] Adamsky A., Kol A., Kreisel T., Doron A., Ozeri-Engelhard N., Melcer T., Refaeli R., Horn H., Regev L., Groysman M. (2018). Astrocytic Activation Generates De Novo Neuronal Potentiation and Memory Enhancement. Cell.

[144] Lamothe-Molina P.J., Franzelin A., Beck L., Li D., Auksutat L., Fieblinger T., Laprell L., Alhbeck J., Gee C.E., Kneussel M. (2020). cFos ensembles in the dentate gyrus rapidly segregate over time and do not form a stable map of space. bioRxiv.

[145] Ivashkina O.I., Gruzdeva A.M., Roshchina M.A., Toropova K.A., Anokhin K.V. (2021). Imaging of C-Fos Activity in Neurons of the Mouse Parietal Association Cortex during Acquisition and Retrieval of Associative Fear Memory. Int. J. Mol. Sci..

[146] Lin X., Itoga C.A., Taha S., Li M.H., Chen R., Sami K., Berton F., Francesconi W., Xu X. (2018). c-Fos mapping of brain regions activated by multi-modal and electric foot shock stress. Neurobiol. Stress..

[147] Mazurkiewicz M., Kambham A., Pace B., Skwarzynska D., Wagley P., Burnsed J. (2022). Neuronal activity mapping during exploration of a novel environment. Brain Res..

[148] Lopez M.R., Wasberg S.M.H., Gagliardi C.M., Normandin M.E., Muzzio I.A. (2024). Mystery of the memory engram: History, current knowledge, and unanswered questions. Neurosci. Biobehav. Rev..

[149] Tanaka K.Z., He H., Tomar A., Niisato K., McHugh T.J. (2018). The hippocampal engram maps experience but not place. Science.

[150] Wang C.H., McHugh T.J. (2023). Putting memories in their place. Cell Res..

[151] Sun X., Bernstein M.J., Meng M., Rao S., Sørensen A.T., Yao L., Zhang X., Anikeeva P.O., Lin Y. (2020). Functionally Distinct Neuronal Ensembles Within the Memory Engram. Cell.

[152] Jiang Y., VanDongen A.M.J. (2021). Selective Increase of Correlated Activity in Arc-Positive Neurons after Chemically Induced Long-Term Potentiation in Cultured Hippocampal Neurons. eNeuro.

[153] Pastuzyn E.D., Day C.E., Kearns R.B., Kyrke-Smith M., Taibi A.V., McCormick J. (2018). The neuronal gene Arc encodes a repurposed retrotransposon Gag protein that mediates intercellular RNA transfer. Cell.

[154] Fitzgerald K.D., Semler B.L. (2009). Bridging IRES elements in mRNAs to the eukaryotic translation apparatus. Biochim. Biophys. Acta.

[155] Hellen C.U. (2009). IRES-induced conformational changes in the ribosome and the mechanism of translation initiation by internal ribosomal entry. Biochim. Biophys. Acta.

[156] Schnatz A., Müller C., Brahmer A., Krämer-Albers E.M. (2021). Extracellular vesicles in neural cell interaction and CNS homeostasis. FASEB Bioadvances.

[157] Ashley J., Cordy B., Lucia D., Fradkin L.G., Budnik V., Thomson T. (2018). Retrovirus-like Gag protein Arc1 binds RNA and traffics across synaptic boutons. Cell.

[158] Erlendsson S., Morado D.R., Cullen H.B. (2020). Structures of virus-like capsids formed by the Drosophila neuronal Arc proteins. Nat. Neurosci..

[159] Jiang Y., Van Dongen A.M. (2020). Neuronal Activity-Dependent Accumulation of Arc in Astrocytes. bioRxiv.

[160] Otmakhov N., Khibnik L., Otmakhova N., Carpenter S., Riahi S. (2004). Forskolin-induced LTP in the CA1 hippocampal region is NMDA receptor dependent. J. Neurophysiol..

[161] Kim D.W., Moon H.C., Lee B.H., Park H.Y. (2024). Decoding Arc transcription: A live-cell study of stimulation patterns and transcriptional output. Learn Mem..

[162] Nikolaienko O., Eriksen M.S., Patil S., Bito H., Bramham C.R. (2017). Stimulus-evoked ERK-dependent phosphorylation of activity-regulated cytoskeleton-associated protein (Arc) regulates its neuronal subcellular localization. Neuroscience.

[163] Gozdz A., Nikolaienko O., Urbanska M., Cymerman I.A., Sitkiewicz E., Blazejczyk M., Dadlez M., Bramham C.R., Jaworski J. (2017). GSK3α and GSK3β Phosphorylate Arc and Regulate its Degradation. Front. Mol. Neurosci..

[164] Hedde P., Malacrida L., Barylko B., Binns D., Albanesi J. (2021). Membrane remodeling by Arc/Arg3.1. Sci. Adv..

[165] Nielsen L., Pedersen C., Erlendsson S., Teilum K. (2019). The Capsid Domain of Arc Changes Its Oligomerization Propensity through Direct Interaction with the NMDA Receptor. Chem. Biol..

[166] Segel M., Lash B., Song J., Ladha A., Liu C.C., Jin X. (2021). Mammalian retrovirus-like protein PEG10 packages its own mRNA and can be pseudotyped for mRNA delivery. Science.

[167] Paolicelli R.C., Bergamini G., Rajendran L. (2019). Cell-to-cell communication by extracellular vesicles: Focus on microglia. Neuroscience.

[168] Tatavarty V., Ifrim M.F., Levin M., Korza G., Barbarese E., Yu J., Carson J.H. (2012). Single-molecule imaging of translational output from individual RNA granules in neurons. Mol. Biol. Cell.

[169] Pinkstaff J.K., Chappell S.A., Mauro V.P., Edelman G.M., Krushel L.A. (2001). Internal initiation of translation of five dendritically localized neuronal mRNAs. Proc. Natl. Acad. Sci. USA.

[170] Jensen S., Thomsen A.R. (2012). Sensing of RNA viruses: A review of innate immune receptors involved in recognizing RNA virus invasion. J. Virol..

[171] Amoyel M., Bach E.A. (2014). Cell competition: How to eliminate your neighbors. Development.

[172] Hamburger V., Levi-Montalcini R. (1949). Proliferation, differentiation and degeneration in the spinal ganglia of the chick embryo under normal and experimental conditions. J. Exp. Zool..

[173] Hamburger V., Levi-Montalcini R. (1951). Selective growth stimulating effects of mouse sarcoma on the sensory and sympathetic nervous system of the chick embryo. J. Exp. Zool..

[174] Baker N.E. (2020). Emerging mechanisms of cell competition. Nat. Rev. Genet..

[175] Madan E., Gogna R., Moreno E. (2018). Cell competition in development: Information from flies and vertebrates. Curr. Opin. Cell Biol..

[176] Banreti A.R., Meier P. (2020). The NMDA receptor regulates competition of epithelial cells in the Drosophila wing. Nat. Commun..

[177] Kim W., Jain R. (2020). Picking winners and losers: Cell competition in tissue development and homeostasis. Trends Genet..

[178] Coelho D.S., Moreno E. (2019). Emerging links between cell competition and Alzheimer’s disease. J. Cell Biol..

[179] Nagata R., Igaki T. (2024). Cell competition: Emerging signaling and unsolved questions. Development.

[180] Valon L., Davidović A., Levillayer F., Villars A., Chouly M., Cerqueira-Campos F., Levayer R. (2021). Robustness of epithelial sealing is an emerging property of local ERK feedback driven by cell elimination. Cell.

[181] Azraq I., Craveiro R.B., Niederau C., Brockhaus J., Bastian A., Knaup I., Neuss S., Wolf M. (2021). Gene expression and phosphorylation of ERK and AKT are regulated depending on mechanical force and cell confluence in murine cementoblasts. Ann. Anat..

[182] Ramiro-Cortes Y., Hobbiss A.F., Israely I. (2013). Synaptic competition in structural plasticity and cognitive function. Philos. Trans. R. Soc. B.

[183] Morciano P., Grifoni D. (2023). Breaking the brain barrier: Cell competition in neural development and disease. Neural Regen. Res..

[184] Yu T., Kuang H., Wu X., Huang Y., Wang J., Wen Z. (2023). Cell competition for neuron-derived trophic factor controls the turnover and lifespan of microglia. Sci. Adv..

[185] Säljö A., Bao F., Shi J., Hamberger A., Hansson H., Haglid K.G. (2004). Expression of c-Fos and c-Myc and Deposition of β-APP in Neurons in the Adult Rat Brain as a Result of Exposure to Short-Lasting Impulse Noise. J. Neurotrauma.

[186] Yamaguchi Y., Miura M. (2015). Programmed Cell Death and Caspase Functions During Neural Development. Curr. Top. Dev. Biol..

[187] Jam F.A., Morimune T., Tsukamura A., Tano A., Tanaka Y., Mori Y., Yamamoto T., Nishimura M., Tooyama I., Mori M. (2020). Neuroepithelial cell competition triggers loss of cellular juvenescence. Sci. Adv..

[188] Sun X., Chen Z., Guo X., Yao M., Johnston L.A., Wu Q. (2023). Stem cell competition driven by the Axin2-p53 axis controls brain size during murine development. Nat. Commun..

[189] Salmina A.B. (2023). Metabolic Plasticity in Developing and Aging Brain. Neurochemistry.

[190] Gilman C.P., Mattson M.P. (2002). Do apoptotic mechanisms regulate synaptic plasticity and growth-cone motility?. Neuromolecular Med..

[191] Salmina A.B., Gorina Y.V., Komleva Y.K., Panina Y.A., Malinovskaya N.A., Lopatina O.L. (2021). Early Life Stress and Metabolic Plasticity of Brain Cells: Impact on Neurogenesis and Angiogenesis. Biomedicines.

[192] Chambers R., Potenza M., Hoffman R., Miranker W. (2004). Simulated Apoptosis/Neurogenesis Regulates Learning and Memory Capabilities of Adaptive Neural Networks. Neuropsychopharmacology.

[193] Shors T.J., Anderson L.M., Curlik D.M., Nokia S.M. (2011). Use it or lose it: How neurogenesis keeps the brain fit for learning. Behav. Brain Res..

[194] Bengzon J., Kokaia Z., Elmér E., Nanobashvili A., Kokaia M., Lindvall O. (1997). Apoptosis and proliferation of dentate gyrus neurons after single and intermittent limbic seizures. Proc. Natl. Acad. Sci. USA.

[195] Martínez-Moreno M., Batlle M., Ortega F.J., Gimeno-Bayón J., Andrade C., Mahy N., Rodríguez M.J. (2016). Diazoxide enhances excitotoxicity-induced neurogenesis and attenuates neurodegeneration in the rat non-neurogenic hippocampus. Neuroscience.

[196] Kittaka M., Mayahara K., Mukai T., Yoshimoto T., Yoshitaka T., Gorski J.P., Ueki Y. (2018). Cherubism Mice Also Deficient in c-Fos Exhibit Inflammatory Bone Destruction Executed by Macrophages That Express MMP14 Despite the Absence of TRAP+ Osteoclasts. J. Bone Miner. Res..

[197] Yoshimura Y., Nakamura K., Seno M., Mochizuki M., Kawai K., Koba S., Watanabe T. (2023). Generation of c-Fos knockout rats, and observation of their phenotype. Exp. Anim..

[198] Kurushima H., Ohno M., Miura T., Nakamura T.Y., Horie H., Kadoya T., Ooboshi H., Kitazono T., Ibayashi S., Iida M. (2005). Selective induction of ΔFosB in the brain after transient forebrain ischemia accompanied by an increased expression of galectin-1, and the implication of ΔFosB and galectin-1 in neuroprotection and neurogenesis. Cell Death Differ..

[199] Tresini M., Lorenzini A., Frisoni L., Allen R.G., Cristofalo V.J. (2001). Lack of Elk-1 phosphorylation and dysregulation of the extracellular regulated kinase signaling pathway in senescent human fibroblast. Exp. Cell Res..

[200] Moreno E., Valon L., Levillayer F., Levayer R. (2019). Competition for Space Induces Cell Elimination through Compaction-Driven ERK Downregulation. Curr. Biol..

[201] Knoepfler P.S., Cheng P.F., Eisenman R.N. (2002). N-myc is essential during neurogenesis for the rapid expansion of progenitor cell populations and the inhibition of neuronal differentiation. Genes. Dev..

[202] Chen J., Guan Z. (2021). Function of Oncogene Mycn in Adult Neurogenesis and Oligodendrogenesis. Curr. Top. Dev. Biol..

[203] Wey A., Knoepfler P.S. (2010). c-myc and N-myc promote active stem cell metabolism and cycling as architects of the developing brain. Oncotarget.

[204] Angeletti P.U., Levi-Montalcini R., Calissano P. (1968). The nerve growth factor (NGF): Chemical properties and metabolic effects. Adv. Enzymol..

[205] Johnston L.A. (2014). Socializing with MYC: Cell competition in development and as a model for premalignant cancer. Cold Spring Harb. Perspect. Med..

[206] Kim J.W., Tchernyshyov I., Semenza G.L., Dang C.V. (2006). HIF-1-mediated expression of pyruvate dehydrogenase kinase: A metabolic switch required for cellular adaptation to hypoxia. Cell Metab..

[207] Guido C., Whitaker-Menezes D., Lin Z., Pestell R.G., Howell A., Zimmers T.A., Casimiro M.C., Aquila S., Ando S., Martinez-Outschoorn U.E. (2012). Mitochondrial fission induces glycolytic reprogramming in cancer-associated myofibroblasts, driving stromal lactate production and early tumor growth. Oncotarget.

[208] Jolivalt C.G., Lee C.A., Beiswenger K.K., Smith J.L., Orlov M., Torrance M.A., Masliah E. (2008). Defective insulin signaling pathway and increased GSK-3 activity in the brain of diabetic mice: Parallels with Alzheimer’s disease and correction by insulin. J. Neurosci. Res..

[209] Böhni R., Riesgo-Escovar J., Oldham S., Brogiolo W., Stocker H., Andruss B.F., Beckingham K., Hafen E. (1999). Autonomous control of cell and organ size by CHICO, a Drosophila homolog of vertebrate IRS1-4. Cell.

[210] Sanaki Y., Nagata R., Kizawa D., Léopold P., Igaki T. (2020). Hyperinsulinemia Drives Epithelial Tumorigenesis by Abrogating Cell Competition. Dev. Cell.

[211] Rardin M.J., Wiley S.E., Naviaux R.K., Murphy A.N., Dixon J.E. (2009). Monitoring phosphorylation of the pyruvate dehydrogenase complex. Anal. Biochem..

[212] Zhou Q., Lam P.Y., Han D., Cadenas E. (2009). Activation of C-Jun-N-Terminal Kinase and Decline of Mitochondrial Pyruvate Dehydrogenase Activity during Brain Aging. FEBS Lett..

[213] Yang D., Wang Y., Qi T., Zhang X., Shen L., Ma J., Pang Z., Lal N.K., McClatchy D.B., Seradj S.H. (2024). Phosphorylation of pyruvate dehydrogenase inversely associates with neuronal activity. Neuron.

[214] Zhou Q., Lam P.Y., Han D., Cadenas E. (2008). c-Jun N-terminal kinase regulates mitochondrial bioenergetics by modulating pyruvate dehydrogenase activity in primary cortical neurons. J. Neurochem..

[215] Papa S., Choy P.M., Bubici C. (2019). The ERK and JNK pathways in the regulation of metabolic reprogramming. Oncogene.

[216] Wilde L., Roche M., Domingo-Vidal M., Tanson K., Philp N., Curry J., Martinez-Outschoorn U. (2017). Metabolic coupling and the Reverse Warburg Effect in cancer: Implications for novel biomarker and anticancer agent development. Semin. Oncol..

[217] Salmina A.B., Okuneva S.O., Taranushenko T.E., Fursov A.A., Prokopenko S.V., Mikhutkina S.V., Malinovskaya N.A., Tagaeva G.A. (2008). Neuron-astroglial interactions in dysregulation of energy metabolism in perinatal ischemic brain damage. Ann. Clin. Exp. Neurol..

[218] De la Cova C., Senoo-Matsuda N., Ziosi M., Wu D.C., Bellosta P., Quinzii C.M., Johnston L.A. (2014). Supercompetitor status of Drosophila Myc cells requires p53 as a fitness sensor to reprogram metabolism and promote viability. Cell Metab..

[219] Morrish F., Noonan J., Perez-Olsen C., Gafken P.R., Fitzgibbon M., Kelleher J., VanGilst M., Hockenbery D. (2010). Myc-dependent mitochondrial generation of acetyl-CoA contributes to fatty acid biosynthesis and histone acetylation during cell cycle entry. J. Biol. Chem..

[220] Morrish F., Hockenbery D. (2014). MYC and mitochondrial biogenesis. Cold Spring Harb. Perspect. Med..

[221] Huang J., Feng Y., Chen X., Li W., Xue L. (2017). Myc inhibits JNK-mediated cell death in vivo. Apoptosis.

[222] Gardner L.B., Lee L.A., Dang C.V. (2002). c-myc Protooncogene. Encycl. Cancer.

[223] Edmunds L.R., Sharma L., Wang H., Kang A., d’Souza S., Lu J., McLaughlin M., Dolezal J.M., Gao X., Weintraub S.T. (2015). c-Myc and AMPK Control Cellular Energy Levels by Cooperatively Regulating Mitochondrial Structure and Function. PLoS ONE.

[224] Bowling S., Di Gregorio A., Sancho M., Pozzi S., Aarts M., Signore M., Schneider M.D., Martinez-Barbera J.P., Gil J., Rodríguez T.A. (2018). P53 and mTOR signalling determine fitness selection through cell competition during early mouse embryonic development. Nat. Commun..

[225] Tamori Y., Deng W.M. (2013). Tissue repair through cell competition and compensatory cellular hypertrophy in postmitotic epithelia. Dev. Cell.

[226] Yoon M.S. (2017). mTOR as a Key Regulator in Maintaining Skeletal Muscle Mass. Front. Physiol..

[227] Sciarretta S., Volpe M., Sadoshima J. (2014). Mammalian target of rapamycin signaling in cardiac physiology and disease. Circ. Res..

[228] Kwon C.H., Luikart B.W., Powell C.M., Zhou J., Matheny S.A., Zhang W., Li Y., Baker S.J., Parada L.F. (2006). Pten regulates neuronal arborization and social interaction in mice. Neuron.

[229] Kleinert M., Sylow L., Fazakerley D.J., Krycer J.R., Thomas K.C., Oxbøll A.J., Jordy A.B., Jensen T.E., Yang G., Schjerling P. (2014). Acute mTOR inhibition induces insulin resistance and alters substrate utilization in vivo. Mol. Metab..

[230] Gwinn D.M., Shaw R.J. (2010). AMPK Control of mTOR Signaling and Growth. Enzymes.

[231] Mannick J.B., Lamming D.W. (2023). Targeting the biology of aging with mTOR inhibitors. Nat. Aging.

[232] Szwed A., Kim E., Jacinto E. (2021). Regulation and metabolic functions of mTORC1 and mTORC2. Physiol. Rev..

[233] Yoon M.S. (2017). The Role of Mammalian Target of Rapamycin (mTOR) in Insulin Signaling. Nutrients.

[234] Ardestani A., Lupse B., Kido Y., Leibowitz G., Maedler K. (2018). mTORC1 Signaling: A Double-Edged Sword in Diabetic β Cells. Cell Metab..

[235] Altas B., Romanowski A.J., Bunce G.W., Poulopoulos A. (2022). Neuronal mTOR Outposts: Implications for Translation, Signaling, and Plasticity. Front. Cell. Neurosci..

[236] Rawat V., Goux W., Piechaczyk M.D., Mello S.R. (2016). c-Fos Protects Neurons Through a Noncanonical Mechanism Involving HDAC3 Interaction: Identification of a 21-Amino Acid Fragment with Neuroprotective Activity. Mol. Neurobiol..

[237] Gallo F.T., Katche C., Morici J.F., Medina J.H., Weisstaub N.V. (2018). Immediate Early Genes, Memory and Psychiatric Disorders: Focus on c-Fos, Egr1 and Arc. Front. Behav. Neurosci..

[238] Ibrahim M.Z.B., Benoy A., Sajikumar S. (2021). Long-term plasticity in the hippocampus: Maintaining within and ’tagging’ between synapses. FEBS J..

[239] Anastacio H.T.D., Matosin N., Ooi L. (2022). Neuronal hyperexcitability in Alzheimer’s disease: What are the drivers behind this aberrant phenotype?. Transl. Psychiatry.

[240] Guzowski J.F., Lyford G.L., Stevenson G.D., Houston F.P., McGaugh J.L., Worley P.F., Barnes C.A. (2000). Inhibition of activity-dependent arc protein expression in the rat hippocampus impairs the maintenance of long-term potentiation and the consolidation of long-term memory. J. Neurosci..

[241] Peineau S., Taghibiglou C., Bradley C., Wong T.P., Liu L., Lu J., Lo E., Wu D., Saule E., Bouschet T. (2007). LTP inhibits LTD in the hippocampus via regulation of GSK3beta. Neuron.

[242] Draffin J.E., Sánchez-Castillo C., Fernández-Rodrigo A., Sánchez-Sáez X., Ávila J., Wagner F.F., Esteban J.A. (2021). GSK3α, not GSK3β, drives hippocampal NMDAR-dependent LTD via tau-mediated spine anchoring. EMBO J..

[243] Duffy D.J., Krstic A., Schwarzl T., Higgins D.G., Kolch W. (2014). GSK3 inhibitors regulate MYCN mRNA levels and reduce neuroblastoma cell viability through multiple mechanisms, including p53 and Wnt signaling. Mol. Cancer Ther..

[244] Alejandre-García T., Kim S., Pérez-Ortega J., Yuste R. (2022). Intrinsic excitability mechanisms of neuronal ensemble formation. eLife.

[245] Campanac E., Gasselin C., Baude A., Rama S., Ankri N., Debanne D. (2013). Enhanced intrinsic excitability in basket cells maintains excitatory-inhibitory balance in hippocampal circuits. Neuron.

[246] Sammari M., Inglebert Y., Ankri N., Russier M., Incontro S., Debanne D. (2022). Theta patterns of stimulation induce synaptic and intrinsic potentiation in O-LM interneurons. Proc. Natl. Acad. Sci. USA.

[247] Márquez L.A., Griego E., López Rubalcava C., Galván E.J. (2023). NMDA receptor activity during postnatal development determines intrinsic excitability and mossy fiber long-term potentiation of CA3 pyramidal cells. Hippocampus.

[248] Redondo R.L., Morris R.G.M. (2011). Making memories last: The synaptic tagging and capture hypothesis. Nat. Rev. Neurosci..

[249] Sossin W.S. (2018). Memory synapses are defined by distinct molecular complexes: A proposal. Front. Synaptic Neurosci..

[250] Kimura F., Itami C. (2019). A Hypothetical Model Concerning How Spike-Timing-Dependent Plasticity Contributes to Neural Circuit Formation and Initiation of the Critical Period in Barrel Cortex. J. Neurosci..

[251] Letzkus J.J., Kampa B.M., Stuart G.J. (2007). Does spike timing-dependent synaptic plasticity underlie memory formation?. Clin. Exp. Pharmacol. Physiol..

[252] Debanne D., Inglebert Y. (2023). Spike timing-dependent plasticity and memory. Curr. Opin. Neurobiol..

[253] Nevian T., Sakmann B. (2006). Spine Ca^2+^ signaling in spike-timing-dependent plasticity. J. Neurosci..

[254] Lube A.J., Ma X., Carlson B.A. (2023). Spike timing-dependent plasticity alters electrosensory neuron synaptic strength in vitro but does not consistently predict changes in sensory tuning in vivo. J. Neurophysiol..

[255] Maheden K., Zhang V.W., Shakiba N. (2022). The Field of Cell Competition Comes of Age: Semantics and Technological Synergy. Front. Cell Dev. Biol..

[256] Marques-Reis M., Moreno E. (2021). Role of cell competition in ageing. Dev. Biol..

[257] Coelho D.S., Schwartz S., Merino M.M.l, Hauert B., Topfel B., Tieche C., Rhiner C., Moreno E. (2018). Culling Less Fit Neurons Protects against Amyloid-β-Induced Brain Damage and Cognitive and Motor Decline. Cell Rep..

[258] Wrigglesworth J., Ward P., Harding I.H. (2021). Factors associated with brain ageing—A systematic review. BMC Neurol..

[259] Sikora E., Bielak-Zmijewska A., Dudkowska M., Krzystyniak A., Mosieniak G., Wesierska M., Wlodarczyk J. (2021). Cellular Senescence in Brain Aging. Front. Aging Neurosci..

[260] Patel M., Antala B., Shrivastava N. (2015). Cell Competition: Roles and Importance as a Central Phenomenon. Crit. Rev. Eukaryot. Gene Expr..

[261] Desjardins S., Mayo W., Vallée M., Hancock D., Le Moal M., Simon H., Abrous D.N. (1997). Effect of aging on the basal expression of c-Fos, c-Jun, and Egr-1 proteins in the hippocampus. Neurobiol. Aging.

[262] Kitraki E., Bozas E., Philippidis H., Stylianopoulou F. (1993). Aging-related changes in IGF-II and c-fos gene expression in the rat brain. Int. J. Dev. Neurosci..

[263] Irwin M., Tare M., Singh A., Puli O.R., Gogia N., Riccetti M., Deshpande P., Kango-Singh M., Singh A. (2020). A Positive Feedback Loop of Hippo- and c-Jun-Amino-Terminal Kinase Signaling Pathways Regulates Amyloid-Beta-Mediated Neurodegeneration. Front. Cell Dev. Biol..

[264] L’Esperance O.J., McGhee J., Davidson G., Niraula S., Smith A.S., Sosunov A.A., Yan S.S., Subramanian J. (2024). Functional Connectivity Favors Aberrant Visual Network c-Fos Expression Accompanied by Cortical Synapse Loss in a Mouse Model of Alzheimer’s Disease. J. Alzheimer’s Dis..

[265] Lu W., Mi R., Tang H., Liu S., Fan M., Wang L. (1998). Over-expression of c-fos mRNA in the hippocampal neurons in Alzheimer’s disease. Neurosci. Lett..

[266] Nandakumar S., Grushko O., Buttitta L.A. (2020). Polyploidy in the adult Drosophila brain. eLife.

[267] Jungas T., Joseph M., Fawal M.-A., Davy A. (2020). Population dynamics and neuronal polyploidy in the developing neocortex. bioRxiv.

[268] Nandakumar S., Rozich E., Buttitta L. (2021). Cell Cycle Re-entry in the Nervous System: From Polyploidy to Neurodegeneration. Front. Cell Dev. Biol..

[269] Choudhury D., Ghosh D., Mondal M., Manna S., Chakraborty S. (2024). Polyploidy and mTOR signaling: A possible molecular link. Cell Commun. Signal..

[270] Esteban-Martínez L., Torres M. (2021). Metabolic regulation of cell competition. Dev. Biol..

[271] Lee H.G., Casadesus G., Nunomura A., Zhu X., Castellani R.J., Richardson S.L., Perry G., Felsher D.W., Petersen R.B., Smith M.A. (2009). The neuronal expression of MYC causes a neurodegenerative phenotype in a novel transgenic mouse. Am. J. Pathol..

[272] Lungu R., Fernandes F.F., Outeiro T.F., Shemesh N. (2022). Brain-wide sensory aberrations in a Parkinson’s Disease mouse model revealed by functional MRI. Neuroscience.

[273] Lanke V., Moolamalla S.T.R., Roy D., Vinod P.K. (2018). Integrative Analysis of Hippocampus Gene Expression Profiles Identifies Network Alterations in Aging and Alzheimer’s Disease. Front. Aging Neurosci..

[274] Bi R., Kong L.L., Xu M., Li G.D., Zhang D.F. (2018). The Arc Gene Confers Genetic Susceptibility to Alzheimer’s Disease in Han Chinese. Mol. Neurobiol..

[275] Schulz L., Ramirez P., Lemieux A., Gonzalez E., Thomson T., Frost B. (2022). Tau-Induced Elevation of the Activity-Regulated Cytoskeleton Associated Protein Arc1 Causally Mediates Neurodegeneration in the Adult Drosophila Brain. Neuroscience.

[276] Zuniga G., Levy S., Ramirez P., De Mange J., Gonzalez E. (2022). Tau-induced deficits in nonsense-mediated mRNA decay contribute to neurodegeneration. Alzheimer’s Dement..

[277] Plagg B., Ehrlich D., Kniewallner K.M., Marksteiner J., Humpel C. (2015). Increased Acetylation of Histone H4 at Lysine 12 (H4K12) in Monocytes of Transgenic Alzheimer’s Mice and in Human Patients. Curr. Alzheimer Res..

[278] Wang H.Y., Lee K.C., Pei Z., Khan A., Bakshi K., Burns L.H. (2017). PTI-125 binds and reverses an altered conformation of filamin A to reduce Alzheimer’s disease pathogenesis. Neurobiol. Aging.

[279] Nagele R.G., D’Andrea M.R., Anderson W.J., Wang H.Y. (2002). Intracellular accumulation of beta-amyloid in neurons is facilitated by the alpha 7 nicotinic acetylcholine receptor in Alzheimer’s disease. Neuroscience.

[280] Alberi L., Liu S., Wang Y., Badie R., Smith-Hicks C., Wu J., Pierfelice T.J., Abazyan B., Mattson M.P., Kuhl D. (2011). Activity-induced Notch signaling in neurons requires Arc/Arg3.1 and is essential for synaptic plasticity in hippocampal networks. Neuron.

[281] Salazar J.L., Yang S., Yamamoto S. (2020). Post-Developmental Roles of Notch Signaling in the Nervous System. Biomolecules.

[282] Müller U., Deller T., Korte M. (2017). Not just amyloid: Physiological functions of the amyloid precursor protein family. Nat. Rev. Neurosci..

[283] Grimm M., Mett J., Stahlmann C., Grösgen S., Haupenthal V. (2017). APP intracellular domain derived from amyloidogenic β- and γ-secretase cleavage regulates neprilysin expression. Front. Aging Neurosci..

[284] Wu J., Petralia R., Kurushima H., Pate H., Jung M. (2011). Arc/Arg3.1 regulates an endosomal pathway essential for activity-dependent beta amyloid generation. Cell.

[285] Rao C.V., Farooqui M., Madhavaram A., Zhang Y., Aschb A.S., Yamada H.Y. (2020). GSK3-ARC/Arg3.1 and GSK3-Wnt signaling axes trigger amyloid-β accumulation and neuroinflammation in middle-aged Shugoshin 1 mice. Aging Cell.

[286] Manago F., Mereu M., Mastwal S., Mastrogiacomo R., Scheggia D., Emanuele M., De Luca M.A., Weinberger D.R., Wang K.H., Papaleo F. (2016). Genetic disruption of Arc/Arg3.1 in mice causes alterations in dopamine and neurobehavioral phenotypes related to schizophrenia. Cell Rep..

[287] Myrum C., Kittleson J., De S., Fletcher B.R., Castellano J., Kundu G., Becker K.G., Rapp P.R. (2020). Survey of the Arc Epigenetic Landscape in Normal Cognitive Aging. Mol. Neurobiol..

[288] Myrum C., Moreno-Castilla P., Rapp P.R. (2022). ’Arc’-hitecture of normal cognitive aging. Ageing Res. Rev..

[289] Micheli L., Creanza T.M., Ceccarelli M., D’Andrea G., Giacovazzo G., Ancona N., Coccurello R., Scardigli R., Tirone F. (2021). Transcriptome Analysis in a Mouse Model of Premature Aging of Dentate Gyrus: Rescue of Alpha-Synuclein Deficit by Virus-Driven Expression or by Running Restores the Defective Neurogenesis. Front. Cell Dev. Biol..

[290] Ding Y., Liu C., Zhang Y. (2023). Aging-related histone modification changes in brain function. iBrain.

[291] Myrum C., Giddaluru S., Jacobsen K., Espeseth T., Nyberg L., Lundervold A.J., Haavik J., Nilsson L.G., Reinvang I., Steen V.M. (2015). Common variants in the ARC gene are not associated with cognitive abilities. Brain Behav..

[292] Subbanna S., Nagre N.N., Umapathy N.S., Pace B.S., Basavarajappa B.S. (2014). Ethanol exposure induces neonatal neurodegeneration by enhancing CB1R Exon1 histone H4K8 acetylation and up-regulating CB1R function causing neurobehavioral abnormalities in adult mice. Int. J. Neuropsychopharmacol..

[293] Shivakumar M., Subbanna S., Joshi V., Basavarajappa B.S. (2020). Postnatal Ethanol Exposure Activates HDAC-Mediated Histone Deacetylation, Impairs Synaptic Plasticity Gene Expression and Behavior in Mice. Int. J. Neuropsychopharmacol..

[294] Subbanna S., Nagre N.N., Shivakumar M., Basavarajappa B.S. (2016). A single day of 5-azacytidine exposure during development induces neurodegeneration in neonatal mice and neurobehavioral deficits in adult mice. Physiol. Behav..

[295] Kempf S.J., Casciati A., Buratovic S., Janik D., Toerne C., Ueffing M., Neff F., Moertl S., Stenerlöw B., Saran A. (2014). The cognitive defects of neonatally irradiated mice are accompanied by changed synaptic plasticity, adult neurogenesis and neuroinflammation. Mol. Neurodegener..

[296] Okuno H., Minatohara K., Bito H. (2018). Inverse synaptic tagging: An inactive synapse-specific mechanism to capture activity-induced Arc/arg3.1 and to locally regulate spatial distribution of synaptic weights. Semin. Cell Dev. Biol..

[297] Choi Y., Cho H., Hoyt C.R., Naegele J.R., Obrietan K. (2008). IGF-1 receptor-mediated ERK/MAPK signaling couples status epilepticus to progenitor cell proliferation in the subgranular layer of the dentate gyrus. Glia.

[298] Liu F., Yang X., Geng M., Huang M. (2018). Targeting ERK, an Achilles’ Heel of the MAPK pathway, in cancer therapy. Acta Pharm. Sin. B.

[299] Ryazanova M.V., Averchuk A.S., Stavrovskaya A.V., Rozanova N.A., Novikova S.V., Salmina A.B. (2023). Arc/Arg3.1 expression in the brain tissues during the learning process in Alzheimer’s disease animal models. Acta Clin. Exp. Neurol..

